# Depth detection limit of a fluorescent object in tissue-like medium with background emission in continuous-wave measurements: a phantom study

**DOI:** 10.1117/1.JBO.29.9.097001

**Published:** 2024-09-02

**Authors:** Goro Nishimura, Takahiro Suzuki, Yukio Yamada, Haruki Niwa, Takuji Koike

**Affiliations:** a Hokkaido University, Research Institute for Electronic Science, Sapporo, Japan; b The University of Electro-Communications, Graduate School of Informatics and Engineering, Chofu, Japan; c The University of Electro-Communications, Center for Neuroscience and Biomedical Engineering, Chofu, Japan

**Keywords:** fluorescence detection, diffuse optics, background emission, variance analysis

## Abstract

**Significance:**

Although the depth detection limit of fluorescence objects in tissue has been studied, reports with a model including noise statistics for designing the optimum measurement configuration are missing. We demonstrate a variance analysis of the depth detection limit toward clinical applications such as noninvasively assessing the risk of aspiration.

**Aim:**

It is essential to analyze how the depth detection limit of the fluorescence object in a strong scattering medium depends on the measurement configuration to optimize the configuration. We aim to evaluate the depth detection limit from theoretical analysis and phantom experiments and discuss the source–detector distance that maximizes this limit.

**Approach:**

Experiments for detecting a fluorescent object in a biological tissue-mimicking phantom of ground beef with background emission were conducted using continuous wave fluorescence measurements with a point source–detector scheme. The results were analyzed using a model based on the photon diffusion equations. Then, variance analysis of the signal fluctuation was introduced.

**Results:**

The model explained the measured fluorescence intensities and their fluctuations well. The variance analysis showed that the depth detection limit in the presence of ambient light increased with the decrease in the source–detector distance, and the optimum distance was in the range of 10 to 15 mm. The depth detection limit was found to be ∼30  mm with this optimum distance for the phantom.

**Conclusions:**

The presented analysis provides a guide for the optimum design of the measurement configuration for detecting fluorescence objects in clinical applications.

## 1 Introduction

Noninvasive measurements of biological systems are crucial in understanding, characterizing, and evaluating the systems. Optical methods are among the most effective ways to obtain noninvasive molecular information about biological and physiological properties. The fluorescence technique, in particular, is a powerful optical technique used to measure sensitively specific biochemical or biophysical properties using specially designed fluorophores in many biological applications, such as pH, [${\mathrm{Ca}}^{2+}$], viscosity, temperature, and their distributions.^1^^,^^2^ However, light scattering limits most fluorescence applications to small-scale applications for microscopic regions or shallow regions of tissue. Near-infrared fluorophores are essential for measuring deeper regions of tissue. However, indocyanine green (ICG) is almost the only choice in near-infrared regions approved by the United States Food and Drug Administration (FDA), and it has been used to visualize the locations of cancer tissue, blood vessels, lymphatic channels, and lymph nodes.^3^^–^^6^ The technique using near-infrared fluorescence cameras is already commercialized and used in clinical environments.^7^^–^^9^ However, these techniques are currently limited to shallow subcutaneous regions, and identifying fluorophores in a region deeper than 10 mm presents a challenge.^10^ This study focuses on the technical aspects of observing a deep fluorescent object.

Examining the distribution of fluorophores is essential for quantifying the fluorescence characteristics of fluorophores in strong scattering media such as biological tissues, and both theoretical and experimental research have a long history.^11^ Numerous studies employing the continuous wave (CW), time, and frequency domain techniques have been conducted. Some of these studies include research on the propagation of fluorescence from fluorescent targets inside the medium^12^^–^^16^ and from homogeneously distributed fluorophores^17^^–^^19^ and research on fluorescence lifetime measurements.^20^^,^^21^ The most advanced and generalized volumetric fluorescence imaging technology is fluorescence diffuse optical tomography and fluorescence molecular tomography, which reconstruct the distribution of fluorophores and fluorescence lifetime in three-dimensional space.^22^^–^^25^

The fluorescence clinical applications *in vivo* in surgical oncology, cardiovascular/cerebrovascular diseases, and other potential fields were reviewed by Refaat et al.^26^ Their paper pointed out that the limitation in penetration depth is a primary obstacle for potential translation and clinical applications and suggested that extending the penetration depth is essential for clinical applications. In addition, many of these applications require fluorescence imaging, which provides more information than only measuring the presence of fluorescence; however, reconstructing fluorescence images of the fluorescence objects in deep tissue requires sophisticated technologies.

Another unique clinical application is assessing the risk of aspiration, which the authors in this study aim to achieve in the future. Elderly people often experience aspiration when food residues in the pyriform sinus (the bilateral laryngeal cavities at the junction of the esophagus and the trachea) unintentionally flow into the trachea. Examining whether food residues remain in the pyriform sinus can evaluate the risk of aspiration. The presence of food residues will be noninvasively monitored using fluorescent foods by irradiating the surface of the neck with excitation light and measuring the presence or absence of fluorescence. The key in this application is how deep targets can be detected because the depth from the front surface to the pyriform sinus is more than 10 mm, much deeper than the targets of many other fluorescence studies.

Fluorescence intensities from fluorescent targets inside media decay almost exponentially with their depths. Therefore, when identifying fluorescent targets at deep positions, a challenge arises from the weak background emission from the media that overlaps with the fluorescence from the target. The fundamental cause of the background is the autofluorescence from endogenous fluorophores in tissues. These fluorophores mostly emit fluorescence excited by ultraviolet to visible light.^27^ So, the autofluorescence excited by near-infrared light becomes very weak. However, even using excitation in the near-infrared region, some endogenous molecules emit autofluorescence, which is not negligible.^28^^–^^30^ In addition, Raman scattering may also cause the background, and such background emission deteriorates the fluorescence signals from fluorescent targets.

Reducing the contribution of the background emission is essential for analyzing the fluorescence data quantitatively. Methods to subtract the contribution of the background emission have been investigated.^31^^–^^33^ Further, methods that actively reduce the contribution, such as multiwavelength imaging and time-resolved techniques, have also been studied.^34^^,^^35^ These techniques enhance the contrast of the fluorescence signals and lead to a much more accurate analysis. However, the detection of the fluorescence targets in deep regions suffers from the fluctuation of the signals, and the contrast alone does not determine the detection limit of depth (depth detection limit). However, until now, the depth detection limit has not been thoroughly studied under the presence of the background emission. Therefore, this study aims to theoretically and experimentally investigate the depth detection limit of a deep fluorescent target in a phantom medium, simulating the human tissue. In addition, the measurements are conducted in the presence of ambient light from environments for clinical applications. Finally, whether the depth detection limit is within the expected depth range of our particular application is determined, and how the source and detector distance can be optimized to improve the depth detection limit toward the measurements for human subjects is identified.

In this study, fluorescence intensity is measured using a photon-counting device capable of detecting weak light, and the fluctuations in the measured weak light are experimentally determined. In particular, we focus on a point illumination and point detection setup for our aspiration study. As a result, it was found that the depth detection limit of the target varies depending on the source–detector (SD) distance and there exists an optimum SD distance at which the depth detection limit is maximized. It was also possible to demonstrate that the depth detection limit increases as the fluctuations in ambient light decrease. The discussion briefly describes the prospective for potential applications of this research, and finally, the conclusions are drawn.

## 2 Theory

**Fig. 1:**
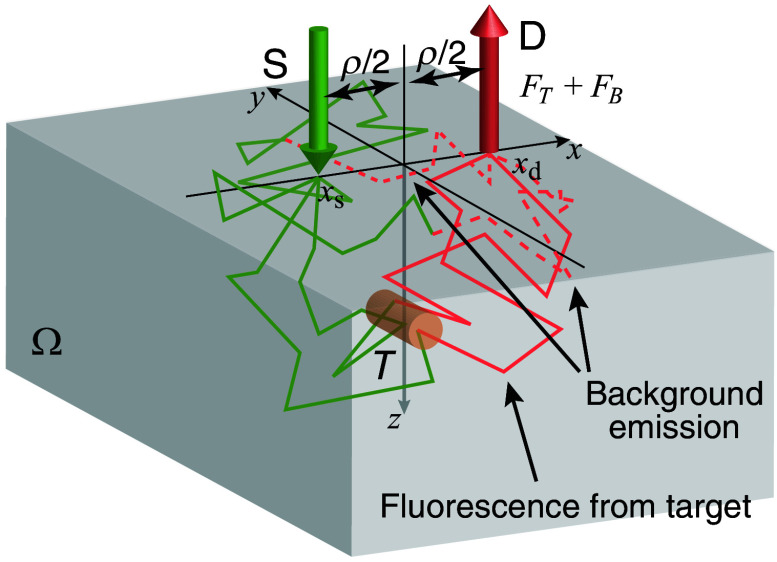
Model for the theoretical analysis. CW excitation light (S) injected at a source position, $x_{s}$, propagates in a homogeneous semi-infinite medium, Ω (green zigzag lines), and a detector (D) at a detection position, $x_{d}$, measures fluorescence from a localized fluorescence target, *T* (solid orange zigzag lines), and homogeneous background emission (broken orange zigzag lines).

We consider a point SD measurement of fluorescence from a fluorescent target (localized fluorophores) embedded in a homogeneous semi-infinite (a half-space) medium, Ω, which emits background fluorescence, as shown in Fig. 1. A CW excitation light at a wavelength of $\lambda_{x}$ is injected at source position $x_{s}$ from the normal direction on the boundary, and the fluorescence light at a wavelength of $\lambda_{m}$ is detected at detector position $x_{d}$ separated from the source by distance ρ. In this study, as we focus on the measurement scheme of the aspiration study, this particular simple setup, in which the target is located under the middle point between the source and the detector to maximize the measured target fluorescence intensity with a fixed SD distance, is analyzed in the theory.

In Secs. 2.1–2.3, we formulate the fluorescence intensities of two processes, the background emission $F_{B}$ and the target fluorescence $F_{T}$, and assume that a simple sum of $F_{B}$ and $F_{T}$ gives the measured fluorescence.

### 2.1 Formula of the Background Emission

First, we consider the background emission similar to the case discussed by Patterson and Pogue^17^ on the homogeneous fluorescence medium. Their results are based on the solution of the photon diffusion equation (DE) under the zero boundary condition. However, one can prove that their result is held in much more general cases, as provided in Sec. 1 in the Supplementary Material. In this study, we employ the solution of the steady-state photon DE under the extrapolated boundary condition in the following derivation. When the medium is illuminated by the unit intensity of the excitation light at $x_{s}$, the background emission intensity at $x_{d}$ is given as

$$F_{B}(\rho )=\frac{\alpha_{B}}{\mu_{ax}-\mu_{am}}[R_{m}(\rho )-R_{x}(\rho )]$$ (1)

and

$$R_{\nu }=\frac{1}{8\pi A_{\nu }D_{\nu }}[\frac{\exp(-{\bar{\mu }}_{\nu }r_{1\nu })}{r_{1\nu }}-\frac{\exp(-{\bar{\mu }}_{\nu }r_{2\nu })}{r_{2\nu }}],$$ (2)

where the subscript ν takes x or m, which represent the excitation and emission wavelengths, $\lambda_{x}$ and $\lambda_{m}$, respectively. $\mu_{ax}$ and $\mu_{am}$ are the absorption coefficients at $\lambda_{x}$ and $\lambda_{m}$, respectively. ${\bar{\mu }}_{\nu }$ is the effective attenuation coefficient (EAC) of the medium defined by ${\overset{¯}{\mu }}_{\nu }=(3\mu_{s\nu }^{\prime }\mu_{a\nu }{)}^{1/2}$, where $\mu_{s\nu }^{\prime }$ is the reduced scattering coefficient. $D_{\nu }$ is the diffusion coefficient, defined by $D_{\nu }=(3\mu_{s\nu }^{\prime }{)}^{-1}$. $\alpha_{B}$ is a proportionality constant, representing the conversion efficiency of the background emission from the excitation light. The proportionality constant $\alpha_{B}$ in Eq. (1) is the product of the quantum efficiency and absorption coefficient of the background fluorophores. However, the origin of the background emission from biological tissues is usually unknown, and thus, $\alpha_{B}$ cannot be estimated as known values. $A_{\nu }$ is the coefficient for the internal reflection due to the Fresnel reflection at the boundary and is approximated by an expression $A_{\nu }=(1+\eta_{\nu })/(1-\eta_{\nu })$, where $\eta_{\nu }=-1.4399n_{\nu }^{-2}+0.7049n_{\nu }^{-1}+0.6681+0.0636n_{\nu }$ and $n_{\nu }$ is the relative refractive index at the boundary.^36^ $r_{1\nu }$ and $r_{2\nu }$ are expressed by $r_{1\nu }=(\rho^{2}+z_{0\nu }^{2}{)}^{1/2}$ and $r_{2\nu }=[\rho^{2}+(z_{0\nu }+2z_{e\nu }{)}^{2}{]}^{1/2}$, respectively, where $z_{0\nu }=(\mu_{s\nu }^{\prime }{)}^{-1}$ is the depth of the virtual isotropic source and $z_{e\nu }=2A_{\nu }D_{\nu }=(2/3)A_{\nu }z_{0\nu }$ is the distance between the true and extrapolated boundaries.

As the reduced scattering coefficient and the refractive index are weakly wavelength-dependent,^37^ we assume them to be constant, i.e., $\mu_{sx}^{\prime }=\mu_{sm}^{\prime }=\mu_{s}^{\prime }$, $D_{x}=D_{m}=D=(3\mu_{s}^{\prime }{)}^{-1}$, $A_{x}=A_{m}=A(n_{x}=n_{m}=n)$, $r_{1x}=r_{1\;m}=r_{1}=(\rho^{2}+z_{0}^{2}{)}^{1/2}$, and $r_{2x}=r_{2\;m}=r_{2}=[\rho^{2}+(z_{0}+2z_{e}{)}^{2}{]}^{1/2}$, where $z_{0}=\mu_{s}^{\prime -1}$ and $z_{e}=(2/3)Az_{0}$.

Equation (2) has a subtraction of two exponential terms having values that are close together, and the substitution of Eq. (2) into Eq. (1) complicates the use of these equations for data analysis. So, we simplify Eqs. (1) and (2) to use them more easily. The details of the simplification are described in Sec. 2 in the Supplementary Material. Briefly, Eq. (2) is expanded into the Taylor series with $\epsilon_{1}=(z_{0}/\rho {)}^{2}$ and $\epsilon_{2}=[(z_{0}+2z_{e})/\rho {]}^{2}$ under the conditions of $\epsilon_{1}\ll 1$ and $\epsilon_{2}\ll 1$ at first, and then using $|({\bar{\mu }}_{m}-{\bar{\mu }}_{x})\rho |\ll 1$, Eq. (1) is approximated to

$$F_{B}(\rho )∼\frac{\alpha_{B}(3+2A)}{4\pi }\frac{\exp(-\bar{\mu }\rho )}{\rho },$$ (3)

where $\bar{\mu }=({\bar{\mu }}_{m}+{\bar{\mu }}_{x})/2$ is the average of the EACs at $\lambda_{x}$ and $\lambda_{m}$. This approximated expression states that the background emission intensity attenuates almost exponentially with the SD distance ρ and inversely states that the average EAC, $\bar{\mu }$, can be determined by measuring the background intensity with varying ρ.

### 2.2 Formula of the Target Fluorescence

Absorption of the excitation light by the target affects the distribution of the excitation light field. As the target, which is located neither in the region near the surface nor close to the source and detector, is significantly smaller than the whole medium volume where the excitation light propagates, we ignore the effect of absorption by the target on the excitation light field, as discussed in our previous paper.^38^ We also employ the analytical solution of the DE under the extrapolated boundary condition, as in many papers in the biomedical optics and photonics community, instead of employing the exact analytical solution,^39^ which has a mathematical complexity in computation, including the complementary error function. Then, we employ the same process as in our previous paper.^24^

The fluorescence emitted from a tiny volume in the target at x=(x,y,z) excited by the source at $x_{s}=(x_{s},y_{s},z_{s})$ is observed at the detector at $x_{d}=(x_{d},y_{d},z_{d})$. The fluence rate of the excitation light field inside the medium $\phi_{x}(x;x_{s})$ is expressed as

$$\phi_{x}(x;x_{s})=\frac{1}{4\pi D_{x}}[\frac{\exp(-{\overset{¯}{\mu }}_{x}l_{1x})}{l_{1x}}-\frac{\exp(-{\overset{¯}{\mu }}_{x}l_{2x})}{l_{2x}}],$$ (4)

where $l_{1x}=[(x_{s}-x{)}^{2}+(y_{s}-y{)}^{2}+(z-z_{0}{)}^{2}{]}^{1/2}$ and $l_{2x}=[(x_{s}-x{)}^{2}+(y_{s}-y{)}^{2}+(z+z_{0}+2z_{e}{)}^{2}{]}^{1/2}$.^12^

The excitation light is absorbed by the fluorophore with the absorption coefficient of $\mu_{af}(x)$, and the absorbed light is partially converted to the fluorescence emission with the quantum efficiency of $\gamma_{f}(x)$. Then, the generated fluorescence emission originating from the position of x propagates to the detector with the probability density function of $\psi_{m}(x_{d};x)$, which is given as

$$\psi_{m}(x_{d};x)=\frac{1}{8\pi AD_{m}}[\frac{\exp(-{\overset{¯}{\mu }}_{m}l_{1m})}{l_{1m}}-\frac{\exp(-{\overset{¯}{\mu }}_{m}l_{2m})}{l_{2m}}],$$ (5)

where $l_{1m}=[(x_{d}-x{)}^{2}+(y_{d}-y{)}^{2}+z^{2}{]}^{1/2}$ and $l_{2m}=[(x_{d}-x{)}^{2}+(y_{d}-y{)}^{2}+(z+2z_{e}{)}^{2}{]}^{1/2}$. When the re-absorption process is negligible, the measured fluorescence will be determined by the sum of fluorescence from all positions in the medium, Ω. Consequently, the fluorescence intensity $F_{T}(x_{d};x_{s})$ is expressed in an integral form as

$$F_{T}(x_{d};x_{s},T)=\int_{\Omega }dx\;\gamma_{f}(x)\mu_{af}(x)\psi_{m}(x_{d};x)\phi_{x}(x;x_{s}),$$ (6)

where T represents the dependence of $F_{T}$ on the distribution of the emission strength of $\gamma_{f}(x)\mu_{af}(x)$ of the fluorophores. When the emission is generated only from a geometrically confined region, we use the term “fluorescence target,” and T represents the geometry of the target.

In our specific problem, the fluorescence target embedded in the medium is a single homogeneous cylinder with a diameter of d, a length of L, an absorption coefficient of $\mu_{T}$, and a quantum efficiency of $\gamma_{T}$. The cylinder is centered at $\left(0,0,z_{T}\right)$, and its longitudinal axis is parallel to the *y*-axis, as shown in Fig. 1. The source and detector are located at $x_{s}=(-\rho /2,0,0)$ and $x_{d}=(\rho /2,0,0)$, respectively. As our experimental parameters were the SD distance ρ and the target depth $z_{T}$, the target signal is given as

$$F_{T}^{\mathrm{cyl}}(\rho ,z_{T})=\alpha_{T}\int_{\Omega }dx\;T_{T}^{\mathrm{cyl}}(x)\psi_{m}(x_{d};x)\phi_{x}(x;x_{s}),$$ (7)

where $\alpha_{T}=\gamma_{T}\mu_{T}$ and a target shape function $T_{T}^{\mathrm{cyl}}(x)$ is expressed as

$$T_{T}^{\mathrm{cyl}}(x)=\{\begin{array}{ll} 1 & \mathrm{if}\;x^{2}+(z-z_{T}{)}^{2}\leq (d{/2)}^{2}\;\mathrm{and}\;y\in [-L/2,L/2]\\ 0 & \mathrm{otherwise}. \end{array}$$ (8)

The integral was calculated numerically.

### 2.3 Measured Signals and a Method for Estimating the Depth Detection Limit

First, we assume that the measured fluorescence intensity is given by the sum of the target fluorescence and background intensities as

$$F^{\mathrm{obs}}(\rho ,z_{T})=F_{T}^{\mathrm{obs}}(\rho ,z_{T})+F_{B}^{\mathrm{obs}}(\rho ).$$ (9)

Here, the superscript “obs” indicates the measured (detected) intensity by the measurement system. The target and background intensities,$F_{T}^{\mathrm{obs}}(\rho ,z_{T})$ and $F_{B}^{\mathrm{obs}}(\rho )$, are supposed to be proportional to the derived functions of Eqs. (7) and (1), respectively. Thus, they are expressed as $F_{T}^{\mathrm{obs}}(\rho ,z_{T})=C_{T}F_{T}^{\mathrm{cyl}}(\rho ,z_{T})$ and $F_{B}^{\mathrm{obs}}(\rho )=C_{B}F_{B}(\rho )$ with the proportionality constants of $C_{T}$ and $C_{B}$, respectively. This simple model is an approximation because the measurements are not monochromatic and have a finite detection area. When the detection area is sufficiently smaller than the SD distance, ρ, the effect of the finite detection area can be included in the proportionality constants. These constants, $C_{T}$ and $C_{B}$, may be theoretically estimated but inaccurate because they depend highly on many unknown factors, such as the detection efficiency, which varies with the specific components used and the measurement conditions. Therefore, we experimentally determined $C_{T}$ and $C_{B}$.

Next, the method for estimating the depth detection limit of the fluorescence target, $z_{T}^{\lim}$, is discussed. In this discussion, we omit the parameters ρ and $z_{T}$ in each expression for brevity unless necessary. First, we introduce the so-called three-sigma rule used in many fields.^40^ The rule presumes that data $x\in \{x_{i}\}\;\;(i=1\cdots N)$ follow a normal distribution, $N(x;\bar{x},\sigma^{2})$, with the variance of $\sigma^{2}$ and the average of $\bar{x}$. Then, if the deviation of data $x_{i}$ from the average is larger than 3σ, $|x_{i}-\bar{x}|>3\sigma$, $x_{i}$ is classified as outside of the original distribution, with a 0.27% chance of misclassification. A similar rule is applied to the estimation of $z_{T}^{\lim}$.

For instance, we consider the condition of $z_{T}$, which satisfies a hypothesis that the average measured intensity of $F^{\mathrm{obs}}$ is significantly larger than the average of the background intensity of $F_{B}^{\mathrm{obs}}$. The validation of this hypothesis is the same as the rejection of the null hypothesis that the average measured intensity of $F^{\mathrm{obs}}$ cannot be distinguished from the average of the background intensity of $F_{B}^{\mathrm{obs}}$. As $F_{T}^{\mathrm{obs}}$ is always positive, only the positive side of the normal distribution, which data obey, is required to estimate the cutoff value, $z_{T}^{\lim}$.

The outputs of the real measurements are the actual data, $M(\rho ,z_{T})$ and $M_{B}(\rho )$, denoting the fluorescence measurement with a target and the background measurement without a target, respectively. $M(\rho ,z_{T})$ and $M_{B}(\rho )$ are statistically distributed around their averages (expectation values) denoted by $F^{\mathrm{obs}}(\rho ,z_{T})$ and $F_{B}^{\mathrm{obs}}(\rho )$, respectively. Assuming that $M-M_{B}$ obeys a normal distribution with the average $E(M-M_{B})$ and the variance $\mathrm{Var}(M-M_{B})$, where E() and Var() represent the average and variance of the data set inside the parenthesis, respectively, the deviation between the averages of $M(\rho ,z_{T})$ and $M_{B}(\rho )$ is standardized as

$$\xi (\rho ,z_{T})=\frac{E(M(\rho ,z_{T})-M_{B}(\rho ))}{[\mathrm{Var}(M(\rho ,z_{T})-M_{B}(\rho )){]}^{1/2}},$$ (10)

and the null hypothesis is rejected when the following inequality is satisfied with the pre-specified significance level α:

$$\xi (\rho ,z_{T})>\xi_{\alpha },\;\mathrm{where}\;\alpha =\int_{\xi_{\alpha }}^{\infty }dxN(x;0,1).$$ (11)

$z_{T}^{\lim}$ is estimated as the cutoff value of $z_{T}$, which satisfies $\xi (\rho ,z_{T}^{\lim})=\xi_{\alpha }$, so $z_{T}^{\lim}$ is given by the solution of the equation

$$F_{T}^{\mathrm{obs}}(\rho ,z_{T})=\xi_{\alpha }[\mathrm{Var}(M(\rho ,z_{T})-M_{B}(\rho )){]}^{1/2},$$ (12)

using $E(M-M_{B})=F^{\mathrm{obs}}-F_{B}^{\mathrm{obs}}=F_{T}^{\mathrm{obs}}$. In the case of $\xi_{\alpha }=3$, Eq. (12) defines the 3σ limit criterion, and in general, the criterion is referred to as the nσ limit criterion.

The current study conducted the measurement using a gated photon-counting method, as explained in the next section. The intensity was expressed as the number of photons counted (photon counts) during a fixed measurement period (bin). The raw count data, $M^{\mathrm{raw}}$, consist of three components: the pure fluorescence intensity from a sample excited by excitation light (on-period), $M_{\mathrm{on}}$; the contamination from the ambient light, $B_{\mathrm{amb}}$; and the dark noise of the detector, $B_{\det}$, i.e., $M^{\mathrm{raw}}=M_{\mathrm{on}}+B_{\mathrm{amb}}+B_{\det}$. To estimate the pure fluorescence intensity, $M_{\mathrm{on}}$, $B_{\mathrm{amb}}+B_{\det}$ is subtracted from $M^{\mathrm{raw}}$ using the measured photon count without the excitation light (off-period) at a slightly different time, $B_{\mathrm{amb}}^{\prime }+B_{\det}^{\prime }$, as

$$M=M_{\mathrm{on}}+B_{\mathrm{amb}}+B_{\det}-[B_{\mathrm{amb}}^{\prime }+B_{\det}^{\prime }].$$ (13)

As the photon count data obey a Poisson distribution, the variance of the data is equal to the average of the data, i.e., Var()=E(). The mean of values obtained by adding or subtracting multiple datasets equals the sum or difference of the means of the original datasets, which follow Poisson distributions. However, the variance of these values equals the sum of variances of the original datasets. When the contamination from the ambient light and the dark noise of the detector are stationary, $E(B_{\mathrm{amb}})=E(B_{\mathrm{amb}}^{\prime })$, $E(B_{\det})=E(B_{\det}^{\prime })$, $\mathrm{Var}(B_{\mathrm{amb}})=\mathrm{Var}(B_{\mathrm{amb}}^{\prime })$, and $\mathrm{Var}(B_{\det})=\mathrm{Var}(B_{\det}^{\prime })$ hold. Finally, the average and the variance of M are expressed as

$$E(M)=E(M_{\mathrm{on}})=F^{\mathrm{obs}},$$ (14)

$$\mathrm{Var}(M)=\mathrm{Var}(M_{\mathrm{on}})+2[\mathrm{Var}(B_{\mathrm{amb}})+\mathrm{Var}(B_{\det})]\;=E(M_{\mathrm{on}})+2[E(B_{\mathrm{amb}})+E(B_{\det})]\;=F^{\mathrm{obs}}+2[E(B_{\mathrm{amb}})+E(B_{\det})].$$ (15)

Applying the same derivation above to $M_{B}$, the variance in Eq. (12) is expressed by

$$\mathrm{Var}(M-M_{B})=F^{\mathrm{obs}}+F_{B}^{\mathrm{obs}}+4[E(B_{\mathrm{amb}})+E(B_{\det})]\;=F_{T}^{\mathrm{obs}}+2F_{B}^{\mathrm{obs}}+4[E(B_{\mathrm{amb}})+E(B_{\det})].$$ (16)

After substituting Eq. (16) into Eq. (12), we have

$$F_{T}^{\mathrm{obs}}(\rho ,z_{T})=\xi_{\alpha }\{F_{T}^{\mathrm{obs}}(\rho ,z_{T})+2F_{B}^{\mathrm{obs}}(\rho )+4[E(B_{\mathrm{amb}})+E(B_{\det})]{\}}^{1/2},$$ (17)

which gives the depth detection limit, $z_{T}^{\lim}$, defined by the nσ limit criterion.

## 3 Experiments

We conducted experiments using a tissue-mimicking phantom to validate the theoretical results and demonstrate the estimation of the depth detection limit of the fluorophore target, $z_{T}^{\lim}$, aiming to design the optimum setup for our aspiration study. As most human subjects in the aspiration study will be older people whose necks slim down and contain less fat, the ground beef phantom is assumed to mimic those necks. Our previous measurements of beef meat evaluated the absorption ($0.023\;{\mathrm{mm}}^{-1}$) and scattering coefficients ($0.92\;{\mathrm{mm}}^{-1}$),^35^ and other work shows similar values.^41^ Those of the human tissue are in a similar range but widely distributed.^42^^,^^43^ Because we aim to determine whether the depth detection limit is in the range of the actual size of the human neck and how the parameters, such as the optical properties and the source–detector distance, affect the limit, we consider the ground beef suitable for our aim. The ground beef was purchased from a supermarket in Chofu city, Japan, and was composed of several unknown cuts of beef meat.

### 3.1 Sample Preparation

**Fig. 2:**
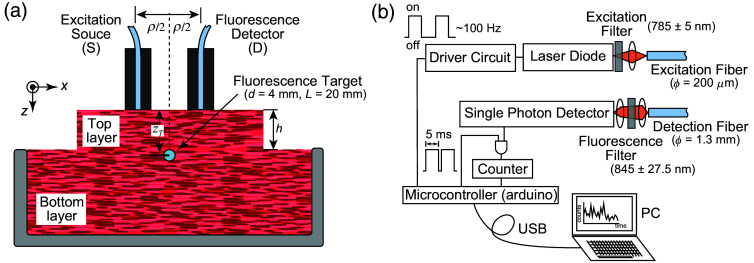
(a) Experimental setup and (b) fluorescence detection device. The phantom consisted of top and bottom layers, and a straw tube containing ICG–milk or milk was embedded at the surface of the bottom layer. The excitation and detection fibers were located on the surface of the top layer, and the fluorescence measurements were conducted by varying the thickness of the top layer and changing the depth of the tube. A photon-counting device measured fluorescence intensities. An on–off-driven excitation laser illuminated the phantom, and the fluorescence photons reaching the detector were counted synchronously with the excitation laser illumination. The photon count data were transferred to a personal computer every 1.6 s.

Figure 2(a) shows our experimental setup using the phantom. The phantom consisted of top and bottom layers of ground beef: the bottom layer (thickness of 25 mm) with a fluorescence target embedded just below its surface and the top layer (thickness, h, varied from 10 to 40 mm). The target was a thin white plastic straw tube (20 mm in length and 4 mm in inner diameter), containing a 0.81-μM ICG–milk solution or milk without ICG. The size of the target was determined to be the expected volume of food residues in the pyriform sinus for future human applications, and by the experimental limitation in making and handling the targets for reproducibility of the measurement results. The depth of the tube is defined as the distance from the top surface to the center of the tube and is expressed by $z_{T}=h+d/2$, which can be varied by changing the thickness of the top layer. ICG was chosen as the fluorophore because it is the most commonly used near-infrared fluorescence dye approved for clinical applications by the FDA. ICG was dissolved in cow milk, selected among various drinkable foods such as soybean milk and yogurt to achieve the best stability and highest fluorescence intensity increase due to an increase in quantum efficiency.^44^ The solution is called ICG–milk hereafter. The preliminary experiment found that the ICG concentration of ∼1  μM gave the maximum fluorescence intensity under similar experimental conditions (see Sec. 3 in the Supplementary Material). The ICG–milk of ∼0.25  ml was capsuled in the straw tubes. The three-dimensional coordinate indicated in Fig. 2(a) is the same as that in Fig. 1, and the fluorescence target of the straw tube was aligned in the same manner as in Fig. 1.

### 3.2 Fluorescence Measurements

Fluorescence intensities were measured by a photon-counting device fabricated in-house, as shown in Fig. 2(b). The device consisted of excitation and fluorescence detection circuits controlled by a microcontroller. The excitation light with the wavelength of 785 nm was generated by a laser diode (L785P090, Thorlabs, Newton, New Jersey, United States) coupled to a single-core multimode fiber (M25L02, Thorlabs) with a laser cleanup filter to reject the spontaneous emission (LD01-785/10, Semrock, Rochester, New York, United States). The average power of the excitation was ∼20  mW at the end of the excitation fiber. The system is categorized as a class 1C device according to IEC 60825-1. The fluorescence emission light was delivered by a bundled fiber (BF13LSMA, Thorlabs), filtered (ET845/55m, Chroma, Bellows Falls, Vermont, United States), and detected by a photon-counting detector (C13366-1350GD, Hamamatsu, Hamamatsu, Japan). The excitation light repeated an on–off cycle at ∼100  Hz, and the fluorescence photons were counted synchronously with the on- or off-period of an exact 5-ms duration. Then, the total photon counts of successive 100 cycles were transferred to a personal computer every 1.6 s, which included the internal processing time. In the post-process, the counts of the off-period were subtracted from the counts of the on-period to cancel the effect of the ambient light and the dark noise of the detector.

The excitation and detector fibers were set on the phantom surface through a very thin transparent plastic film with the SD distance, ρ, in the same manner as that in Fig. 2(a). First, we made a phantom with the target not containing ICG (only milk) and measured the background signal from the phantom with varying ρ and $z_{T}$. Then, we measured the fluorescence signals from the phantom with the target containing ICG–milk in the same manner.

The photon counter ran continuously during the measurements, and three to four consecutive measurement data were averaged for the following data analysis. Hereafter, the fluorescence intensities are given as the photon counting rates [counts per bin (cpb)] with a counting bin size of 0.5 s (5  ms×100). In the measurements, the counts of the raw background data before taking the difference between the on-period and off-period of the laser illumination were ∼500 to 900 kcpb, mostly due to the ambient light. The dark count of the detector was ∼1.25  kcpb.

## 4 Results

**Fig. 3:**
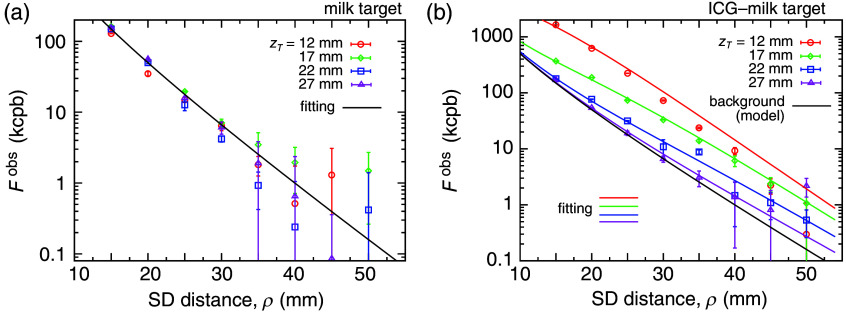
Fluorescence intensities with the (a) milk-only target and (b) ICG–milk target. The abscissa is the SD distance, ρ, and the different colors indicate the different target depths, $Z_{T}=12$, 17, 22, and 27 mm (top layer thicknesses, d=10, 15, 20, and 25 mm). The fitting of Eq. (18) to the background intensities is shown by black solid curves. The fittings of Eq. (19) to the fluorescence intensities with the ICG–milk target are shown by colored solid curves in panel (b).

Figure 3 summarizes the measurement results. Symbols and error bars show the average fluorescence intensities and their fluctuations, respectively, with different colors corresponding to the target depths, $z_{T}$. The fluorescence intensities with the milk-only target are shown in Fig. 3(a). Although the emission from the ground beef with a milk-only target may not solely originate from a fluorescence process, we use the term “fluorescence” throughout and do not distinguish among the possible origins. The intensities in Fig. 3(a) almost exponentially decrease with the SD distance, ρ, and irregularly vary at the large SD distances (ρ>35 mm) because the fluctuation level of the ambient light from the environment was close to that of the fluorescence intensities from the phantom. The target depth, $z_{T}$, did not affect the intensities as there was no systematic dependence on $z_{T}$. Therefore, the fluorescence intensities with the milk-only target can be considered to be the background intensities from the unknown origin of the ground beef.

The measured fluorescence intensities from the ground meat with the ICG–milk target are shown by symbols in Fig. 3(b), and these fluorescence intensities strongly depend on both the depth of the target, $z_{T}$, and SD distance, ρ, in contrast to those with the milk-only target. For comparison, the background intensities discussed later are also shown by a black solid curve in Fig. 3(b). The intensities with the ICG–milk target at $z_{T}=12,17$, and 22 mm are larger than the background intensities with ρ up to 40 to 45 mm. By contrast, the intensities at $z_{T}=27\;\mathrm{mm}$ are very close to the background intensities.

The approximated theoretical expression of the background intensity, Eq. (3), has two unknown parameters, $\bar{\mu }$ and $\alpha_{B}$. In addition, the measured intensities are proportional to the measurement efficiency determined by the measurement device. Therefore, Eq. (3) is modified as

$$F_{B}^{\mathrm{obs}}(\rho )={\tilde{C}}_{B}\frac{\exp(-\bar{\mu }\rho )}{\rho },$$ (18)

where ${\tilde{C}}_{B}$ is a constant defined as ${\tilde{C}}_{B}=C_{B}\alpha_{B}(3+2A)/(4\pi )$ to be used in the fitting analysis. Figure 3(a) shows the result of the $\chi^{2}$ fitting of Eq. (18) to all measured data by the black solid curve and indicates that the background intensities are well expressed by Eq. (18) with $\bar{\mu }=0.161\pm 0.009\;{\mathrm{mm}}^{-1}$ and ${\tilde{C}}_{B}=(2.50\pm 0.34)\times 10^{4}$ kcpb ($\chi^{2}/n=31.6$, the average weighted residual = −0.55, $R^{2}=0.99$).

Assuming that $\mu_{sx}^{\prime }=\mu_{sm}^{\prime }=1\;{\mathrm{mm}}^{-1}$, the average absorption coefficient ${\bar{\mu }}_{a}=(\mu_{ax}+\mu_{am})/2$ is estimated as $0.0086\;{\mathrm{mm}}^{-1}$. This value of ${\bar{\mu }}_{a}$ is smaller than $0.023\;{\mathrm{mm}}^{-1}$ measured using a beef meat block by a time-of-flight method at 780 nm.^35^ The scattering coefficient of beef meat varies by approximately a factor of 2 depending on the part of the beef meat.^41^ Considering this variation in the scattering coefficient, the estimated value of ${\bar{\mu }}_{a}$ has the same range of variation because the measurements only allow for the estimation of the EAC. In addition, ground beef was a mixture of different parts of beef meat; in particular, it contained more fat and connecting tissue with much less myoglobin, but with an unknown composition. Therefore, the difference from the beef meat block may be caused by the composition.

Equation (9) is valid, assuming that the background emission process and the target’s fluorescence process do not couple. Considering $F_{T}^{\mathrm{obs}}(\rho ,z_{T})=C_{T}F_{T}^{\mathrm{cyl}}(\rho ,z_{T})$ for a cylindrical target, Eq. (9) is modified to

$$F^{\mathrm{obs}}(\rho ,z_{T})=F_{T}^{\mathrm{obs}}(\rho ,z_{T})+F_{B}^{\mathrm{obs}}(\rho )={\tilde{C}}_{T}F_{T}^{\mathrm{cyl}}(\rho ,z_{T})/\alpha_{T}+F_{B}^{\mathrm{obs}}(\rho ),$$ (19)

where ${\tilde{C}}_{T}=\alpha_{T}C_{T}$. Here, the result of the fitting shown in Fig. 3(a) can be used for $F_{B}^{\mathrm{obs}}$ in Eq. (19). The values of $F_{T}^{\mathrm{cyl}}/\alpha_{T}$ for a particular combination of ρ and $z_{T}$ were calculated using $\mu_{ax}=\mu_{am}=0.0086\;{\mathrm{mm}}^{-1}$, $\mu_{s}^{\prime }=1\;{\mathrm{mm}}^{-1}$, and n=1.37. Then, Eq. (19) was fit to all fluorescence intensities to minimize the weighted $\chi^{2}$, yielding ${\tilde{C}}_{T}=3.65\times 10^{7}$ kcpb·mm ($\chi^{2}/n=173$, the average weighted residual = −1.36, $R^{2}=0.99$). The colored solid curves in Fig. 3(b) show the fitting results, and the black solid curve shows the background intensity of $F_{B}^{\mathrm{obs}}(\rho )$ determined in Fig. 3(a) for comparison. The model function, Eq. (19), agrees well with the fluorescence intensities except for $z_{T}=12\;\mathrm{mm}$ (red solid curve) and some data points below 2 kcpb, using only the single scaling constant, ${\tilde{C}}_{T}$. The discrepancies between the fitting and the data with $z_{T}=12\;\mathrm{mm}$ were caused by the inaccuracies of the measurement configuration, such as the flatness of the phantom surface, the depth of the target, and the positioning of the source and detector. These configuration errors more significantly affected the results for the shallower target. In addition, the data points deviate from the fitting curves more than the error bars due to insufficient data points to estimate the errors. The data have large relative variations in the lowest signal range below 2 kcpb, where the measured data were unreliable due to the fluctuation of the photon counts. However, except for $z_{T}=12\;\mathrm{mm}$, no systematic deviation can be seen. The values of $\chi^{2}/n$ and the average weighted residual calculated only with $z_{T}=17-27\;\mathrm{mm}$ became 23.3 and 2.6, respectively, indicating that the model agrees well with the data except for the data with $z_{T}=12\;\mathrm{mm}$. Therefore, ${\tilde{C}}_{T}$ is independent of ρ, and $z_{T}$ is consistent with this result. It is worth noting that the difference between the black (background) and purple ($z_{T}=27\;\mathrm{mm}$) solid curves in Fig. 3(b) becomes large with the increase in the SD distance, indicating that the larger SD distance is more effective in detecting fluorescence from a deep target if there is no variation of the measured data.

**Fig. 4:**
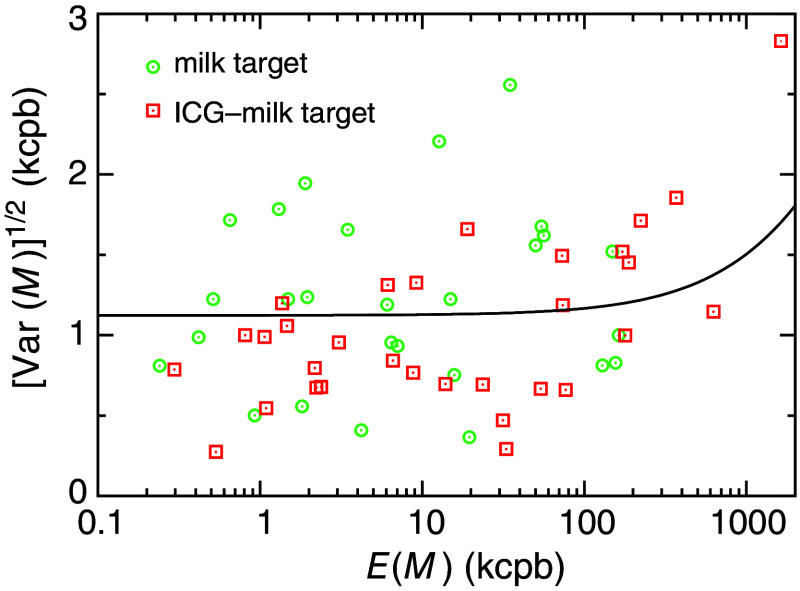
Standard deviation of the measured photon counts, $[\mathrm{Var}(M){]}^{1/2}$, as a function of their averages, E(M). Each symbol corresponds to the pair of E(M) and $[\mathrm{Var}(M){]}^{1/2}$ of the measured data from three to four successive photon count data for the various $z_{T}$ of the milk-only (green circles) or ICG–milk (red squares) targets. The black solid curve indicates $[\mathrm{Var}(M){]}^{1/2}$ obtained by fitting Eq. (15) to the whole data.

For estimating the depth detection limit, $z_{T}^{\lim}$, we first analyze the average and standard deviation of the measured photon counts, M, from three to four successive photon count data. Figure 4 shows the standard deviation of the measured photon counts, $[\mathrm{Var}(M){]}^{1/2}$, as a function of the average, E(M), indicating no significant correlations between E(M) and $[\mathrm{Var}(M){]}^{1/2}$. The uncorrelated relationship means that the emission from the background and the target did not dominantly determine the variation. There was no significant difference between the milk-only and the ICG–milk samples, indicating that the contributions of the ambient light and the dark noise of the detector were the same for the measurements with the milk-only and the ICG–milk samples. As the photon count data obey a Poisson distribution, Var(M) is given by Eq. (15), which was fit to the whole data in Fig. 4 and is shown by the black solid curve. From the fitting, $E(B_{\mathrm{amb}})+E(B_{\det})=(6.3\pm 0.8)\times 10^{2}$ kcpb was obtained ($\chi^{2}/n=0.36$ kcpb). The average residual of the fitting was 0.0077, indicating that the model did not systematically deviate from the data.

The measured raw photon counts, $M^{\mathrm{raw}}$, ranged from 500 to 900 kcpb, which were very close to the value of $E(B_{\mathrm{amb}})+E(B_{\det})$ estimated above. This value is significantly larger than the photon counts of the background and target intensities shown in Fig. 3. These large raw photon counts were mainly attributed to the ambient light because the dark counts of the detector, $E(B_{\det})$, were negligibly small, ∼1.25  kpcb. As a result, the variation of the ambient light intensities during the measurements must have obscured the relationship between $E(F^{\mathrm{obs}})$ and $[\mathrm{Var}(F^{\mathrm{obs}}){]}^{1/2}$, resulting in no significant correlations between E(M) and $[\mathrm{Var}(M){]}^{1/2}$, as shown in Fig. 4.

**Fig. 5:**
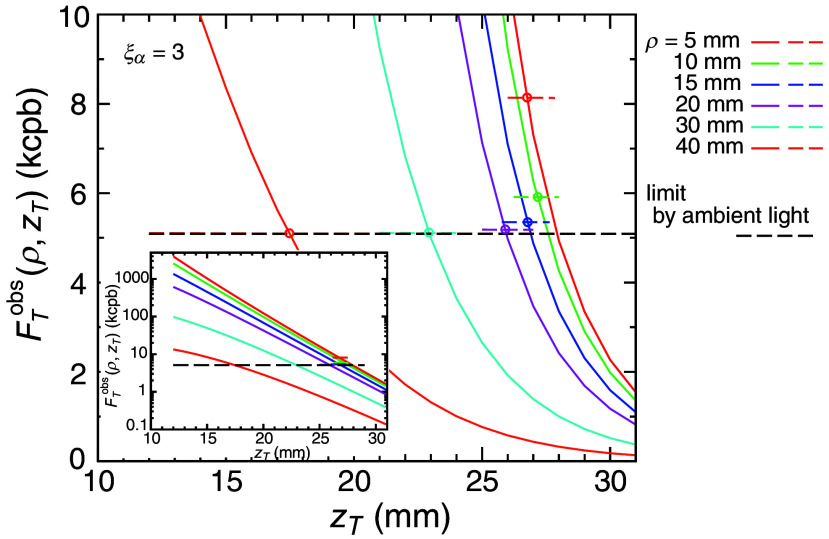
Target fluorescence intensities as a function of the target depth, $z_{T}$, and the threshold calculated by Eq. (17) with $\xi_{\alpha }=3$ (the 3σ limit). Solid curves are the target fluorescence intensities, and the different colors indicate the different SD distances, ρ. The dashed line segments show the thresholds. The black dashed line indicates the threshold when the background emission is ignored, i.e., $F_{B}^{\mathrm{obs}}=0$. The small circles indicate the intersection points where Eq. (17) is satisfied for each SD distance, i.e., the depth detection limit $z_{T}^{\lim}$.

Now, we estimate the detection limit of the target, $z_{T}^{\lim}$, in our phantom system using Eq. (17) with the experimentally determined values of $F_{T}^{\mathrm{obs}}$, $F_{B}^{\mathrm{obs}}$, and $E(B_{\mathrm{amb}})+E(B_{\det})$. Solid curves in Fig. 5 show the target fluorescence intensities, $F_{T}^{\mathrm{obs}}(\rho ,z_{T})$, which is in the left side of Eq. (17), as a function of $z_{T}$, and the dashed line segments in Fig. 5 is the thresholds determined by the right side of Eq. (17) with $\xi_{\alpha }=3$. Different colors denote different ρ, and the black dashed line is the threshold for the case in which the background fluorescence is ignored. The inset of Fig. 5 shows the whole profiles of $F_{T}^{\mathrm{obs}}$ in a logarithmic scale. The target intensities, $F_{T}^{\mathrm{obs}}$, show an approximately exponential decrease with the increase in $z_{T}$ as shown in the inset of Fig. 5. The thresholds for large ρ (ρ>20 mm) are very close to the black dashed line due to the strong contribution from the ambient light.

The small circles in Fig. 5 indicate the intersection points where Eq. (17) is satisfied for each ρ. As ρ increases, the intersection point moves slightly to smaller values of $z_{T}$ up to ρ=20  mm and then drops significantly, showing that the depth sensitivity is not much improved with the increase in ρ. The change up to 20 mm is reasonable because the strong ambient light was dominant in the fluctuation of the measured fluorescence intensities, and the change in the intensities with changing ρ had a minor effect on the variance of the intensities. The target detectability worsens, particularly for ρ larger than 20 mm, because $F_{T}^{\mathrm{obs}}$ decreases significantly and is buried under the fluctuation of the ambient light. Under the current experimental condition, $z_{T}^{\lim}$ does not exceed ∼27  mm with any SD distance, ρ.

**Fig. 6:**
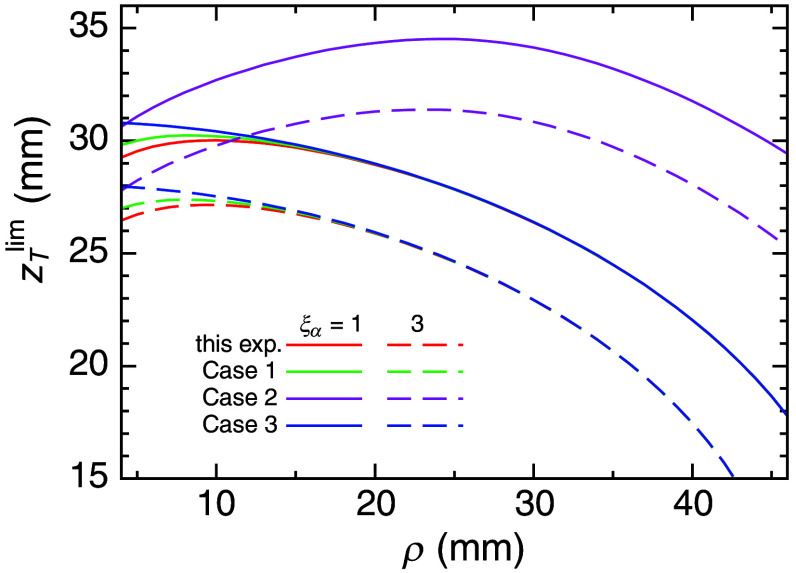
Depth detection limit, $z_{T}^{\lim}$, as a function of the SD distance, ρ, with $\xi_{\alpha }=1$ and 3 indicated by the solid and dashed curves, respectively. The conditions and colors of the four cases are listed in Table 1.

Figure 6 shows $z_{T}^{\det}$ as a function of ρ. The values of $z_{T}^{\det}$ were numerically calculated as the intersection points illustrated in Fig. 5. Three cases in addition to the experiment in this study (case 0) are listed in Table 1 and included in Fig. 6. Cases 0 to 3, indicated by different colors, presume different background fluorescence as well as the presence or absence of the ambient light, with the parameter, $\xi_{\alpha }$, taking 1 (solid curves) and 3 (dashed curves) for all cases. In cases 1 and 2, the background fluorescence is set as 50% of that in the ground beef experiment in this study by considering the results of preliminary measurements of human necks. Case 2 assumes no ambient light, whereas case 3 assumes no background emission.

**Table 1:** Four cases in Fig. 6.

Cases	$F_{B}^{\mathrm{obs}}$	$B_{\mathrm{amb}}$ and $B_{\det}$	$\xi_{\alpha }$	Color in Fig. 6
0 (this experiment)	100% as was measured	$B_{\mathrm{amb}}+B_{\det}$	1, 3	Red
1	50%	$B_{\mathrm{amb}}+B_{\det}$		Green
2	50%	$B_{\det}$		Purple
3	0%	$B_{\mathrm{amb}}+B_{\det}$		Blue

An increase in $\xi_{\alpha }$ decreases $z_{T}^{\lim}$, and the change in $\xi_{\alpha }$ from 1 to 3 decreases $z_{T}^{\lim}$ by 4 to 5 mm. The background emission is found to affect $z_{T}^{\lim}$ for small ρ (ρ<15  mm) by comparing case 0 (100% $F_{B}^{\mathrm{obs}}$) with case 1 (50% $F_{B}^{\mathrm{obs}}$) and case 3 (0% $F_{B}^{\mathrm{obs}}$). By contrast, it was found that the ambient light significantly reduced $z_{T}^{\lim}$ by comparing case 1 (with $B_{\mathrm{amb}}$) with case 2 (without $B_{\mathrm{amb}}$). Note that the dark noise of the detector, $B_{\det}$, is negligible compared with the ambient light, $B_{\mathrm{amb}}$.

The optimum SD distance, $\rho^{\mathrm{opt}}$, yielding the maximum $z_{T}^{\lim}$, strongly depends on the cases. In case 2 (with $F_{B}^{\mathrm{obs}}$ but without $B_{\mathrm{amb}}$), $z_{T}^{\lim}$ initially increases with the increase in ρ but then decreases. The initial increase indicates that the background emission, $F_{B}^{\mathrm{obs}}$, decreases with the increase in ρ more quickly than the target fluorescence, $F_{T}^{\mathrm{obs}}$, for small ρ. In this case, $l_{1x}$, $l_{2x}$, $l_{1m}$, and $l_{2m}$ in Eqs. (4) and (5) are determined by $z_{T}$, resulting in the target fluorescence being approximated by a finite order of a polynomial of ρ instead of the exponential function for the background emission. Consequently, the background emission decreases more significantly with increasing ρ. On the other hand, for large ρ, $l_{1x}$, $l_{2x}$, $l_{1m}$, and $l_{2m}$ change dominantly by ρ. The product $\phi_{x}\psi_{m}$ inside the integral of Eq. (6) decreases much faster than exp(−μρ)/ρ with increasing ρ, at least considering a sufficiently small target. As a result, the target fluorescence decreases faster than the background emission with the increase in ρ. For large ρ, the detector noise also limits the detection of the target fluorescence. Therefore, ρ of ∼25  mm provides the maximum $z_{T}^{\lim}$ of 34 mm and 31 mm for $\xi_{\alpha }=1$ and 3, respectively, as shown in Fig. 6.

For cases 0, 1, and 3, three curves are indistinguishable at ρ>15  mm in Fig. 6, indicating that $z_{T}^{\lim}$ is independent of $F_{B}^{\mathrm{obs}}$. As the dark noise of the detector, $B_{\det}$, was negligibly small, the ambient light determines the decrease in $z_{T}^{\lim}$ with the increase in ρ due to decreasing the fluorescence target intensity. The minor differences in the curves are seen at ρ<15  mm, and the background emission, $F_{B}^{\mathrm{obs}}$, causes a decrease in $z_{T}^{\lim}$ with a decrease in ρ. The increase in the background emission with the decrease in ρ is more significant than the increase in the target fluorescence, as explained above. Case 3 is an extreme case without the presence of the background emission, indicating that a smaller ρ always improves the detectability of the target.

**Fig. 7:**
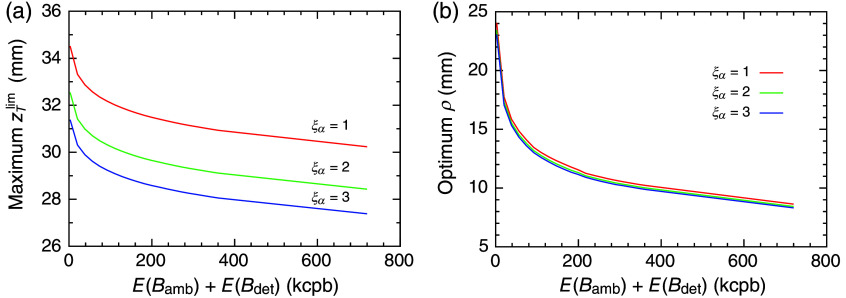
(a) Depth detection limit, $z_{T}^{\lim}$. (b) Optimum SD distance, $\rho^{\mathrm{opt}}$, as a function of $E(B_{\mathrm{amb}})+E(B_{\det})$ with varying $\xi_{\alpha }$ for case 1. Red, green, and blue colors indicate $\xi_{\alpha }=1,\;2$, and 3, respectively. Because $E(B_{\det})$ was estimated at ˜1.25kcpb, the abscissa is almost equal to $E(B_{\mathrm{amb}})$.

To visualize more about the effect of the ambient light, Figs. 7(a) and 7(b) show how the ambient light affects $z_{T}^{\lim}$ and $\rho^{\mathrm{opt}}$, respectively, for case 1. As expected, the maximum $z_{T}^{\lim}$ increases with the decrease in $E(B_{\mathrm{amb}})+E(B_{\det})$ in Fig. 7 and with the decrease in $\xi_{\alpha }$. In particular, reducing $E(B_{\mathrm{amb}})+E(B_{\det})$ to less than 100 kcpb is very effective in improving the detectability. Reducing ambient light is most effective in the current experiment setup because $B_{\det}$ is negligible. Still, in general, the choice of the detector is also important for measuring the deep fluorescent target. Figure 7(b) shows that the optimum SD distance, $\rho^{\mathrm{opt}}$, decreased with the increase in $E(B_{\mathrm{amb}})+E(B_{\det})$, but the change in $\rho^{\mathrm{opt}}$ with the change in $\xi_{\alpha }$ is tiny. In particular, both the changes in $\rho^{\mathrm{opt}}$ and the maximum $z_{T}^{\lim}$ are small at large values of $E(B_{\mathrm{amb}})+E(B_{\det})$, suggesting that the SD distance around 10 mm is not so critical to improving the measurements of a deep fluorescent target with the presence of the ambient light. The results also indicate that an extension of $z_{T}^{\lim}$ of more than 35 mm is very difficult, and the target depth of ∼30  mm is the practical detection limit.

## 5 Discussion

The experimental results with the ground meat phantom are well explained by the model equations derived in Sec. 2, and the SD distance dependence of the background emission determined the EAC of the ground meat. Then, these results determined the maximum depth detection limit and the optimum SD distance. Preliminary measurements of the background emission from healthy male human subjects aged 22 to 70 (N=3) without the intake of ICG–milk were conducted using the same fluorescence detection system as in the phantom study to compare the background emission with that of the phantom. The optical probe shown in Fig. 2 was in contact with the neck skin with ρ being varied from 15 to 35 mm. The measurements on humans were approved by the Ethics Committee of the University of Electro-Communications, and written informed consent from the subjects was obtained.

The background emission from the human neck tissue with varying ρ was analyzed in the same manner as the phantom experiment, determining $\overset{¯}{\mu }=0.129$ to $0.235\;{\mathrm{mm}}^{-1}$, which is close to the result of the ground meat ($\overset{¯}{\mu }=0.161\;{\mathrm{mm}}^{-1}$). Their background intensities were about half of the intensities of the ground meat, which corresponded to cases 1 and 2 in Sec. 4. Contamination by ambient light is not avoidable in clinical environments. So, case 1 roughly mimics the real environment for clinical applications. From the results for case 1 in Fig. 6, the maximum of $z_{T}^{\lim}$ is estimated as 28 and 31 mm for $\xi_{\alpha }$ of 3 and 1, respectively, and the optimum SD distance, $\rho^{\mathrm{opt}}$, is ∼10 to 15 mm. If the ambient light can be suppressed, $z_{T}^{\lim}$ increases by ∼5  mm, with extending $\rho^{\mathrm{opt}}$ ∼25  mm, as in case 2 in Fig. 6.

The previous study employing the time–domain measurement introduced the contrast analysis for determining the optimum SD distance under ideal conditions with no ambient light.^35^ In the time–domain measurements, an appropriate temporal window, which selects the light paths, can reduce the background emission and can improve the contrast of the target fluorescence. The null SD distance (ρ=0) most effectively improves the contrast. By contrast, all light paths contribute to the background emission in the CW measurements. In particular, the paths in a shallow region above the target contribute more strongly than those in a deep region, including the target, when ρ becomes short. As ρ increases, the contribution of a shallow region compared with that of the target region decreases first, but it increases further as ρ increases. Therefore, $\rho^{\mathrm{opt}}$ exists somewhere between very short ρ and very long ρ, as shown for case 2 in Fig. 6. The time–domain methods may have the margin to enhance depth detectability by more appropriately selecting the temporal window at the cost of a more complex system and expenses.

The unavoidable causes of the background emission are autofluorescence and Raman scattering originating from the medium itself, which limit the depth detectability in an ideal condition. It is known that autofluorescence and Raman scattering reduce in a wavelength range longer than that used in this study, and some studies suggest using the tail of the fluorescence spectrum of ICG or using ICG derivatives.^45^^,^^46^ However, extending to a longer wavelength range has some difficulties in practice. As the wavelength increases, the fluorescence intensity of ICG diminishes, and the silicon (Si)-based detector loses its sensitivity. Indium gallium arsenide (InGaAs) detectors are alternatives to Si-based detectors beyond 900 nm. However, InGaAs detectors suffer from large dark counts, and their active areas are small due to the trade-off between the dark counts and the detection efficiency. The large dark counts degrade the depth sensitivity, as shown in Fig. 7. Therefore, using the longer wavelength range may not help improve the depth sensitivity under the current device technology.

The results obtained here suggest a possibility of detecting the fluorescence from a significantly deep location up to ∼30  mm. For the clinical application to the pulmonary aspiration risk assessment, it is necessary to detect the fluorescence from the fluorescent foods in the pyriform sinus at the front surface of the neck. To consider the difference in the optical properties between the phantom and the human tissue, the depth detection limits were simulated when the EAC is square root of two times that of the phantom as described in Sec. 4 in the Supplementary Material. Square root of two times EAC, corresponding to doubled $\mu_{ax}$ and $\mu_{am}$ or doubled $\mu_{s}^{\prime }$, makes the depth detection limit 6 to 7 mm shallower. The absorption and the scattering coefficients have large varieties in bovine tissues^41^ and human tissues,^42^^,^^43^ depending on the individuals.

The phantom was also simplified, although the human neck is curved and consists of different tissue layers and structures such as the airway. Further, the model in the analysis assumed a simple semi-infinite space. It is possible to extend the model to the case of a curved surface, which is a very interesting problem to be solved in the future. As shown in Fig. 3, the fluorescence signals are measurable in the range of the SD distance of ∼30  mm. On the other hand, the adult neck size is more than 100 mm. Therefore, we assume the infinite plane surface as the first-order approximation of the curved surface of the neck. In this paper, we intended not to make a precise phantom and model for the human neck but to provide useful information for future human measurements. We think that the actual depth detection limit of the individual needs to be tested by human subjects.

The depth of the pyriform sinus from the neck surface and the optical properties of the neck change from person to person. However, most patients having aspiration risk are elderly people, and the muscles and fat in their neck usually slim down (presumably thickness is in the range of ∼20 to 30 mm), resulting in shallower pyriform sinus and smaller effective attenuation coefficients than those for healthy or young people. Thus, it is highly expected that the fluorescence from the pyriform sinus of elderly people is detectable except in patients with excessive obesity. Therefore, we believe that this study encourages clinical tests of the method.

In clinical applications, the concentration of ICG must be optimized. In this study, ∼1  μM of ICG was used in the experiments because this concentration gave the highest fluorescence intensity, as shown in Sec. 3 in the Supplementary Material. By contrast, the published ICG concentration value in oral administration of ICG for humans was much larger than the value used in this study.^47^ As the fluorescence intensity depends on the geometry and shape of the fluorescence target due to the inner filter effect, the optimum concentration of ICG for detecting remaining foods in the pyriform sinus may not be the same value. For example, the depth detection limit can be improved by ∼2  mm if the fluorescence intensity is doubled by the increase in the ICG concentration, as shown in Sec. 4 in the Supplementary Material.

The excitation power in the experiment was 20 mW. For medical purposes, this power range is categorized as class 1C and has a margin to be increased. Figure S4 in the Supplementary Material shows the simulation results with the doubled excitation power. The depth detection limit increases ∼2  mm. The improvement is gradual but also possible. Therefore, the optimizations of the excitation power and the concentration of ICG are also needed for the realization of the noninvasive method of evaluating the risk of pulmonary aspiration.

## 6 Conclusion

We conducted experimental and theoretical studies to estimate the depth detection limit of CW fluorescence from a fluorescent target deeply embedded in a medium, which emits the background fluorescence. The analytical expressions based on the photon diffusion equation were provided for the background fluorescence from a homogeneous semi-infinite medium and the fluorescence from a fluorescent target embedded in the medium as functions of the SD distance and the depth of the target. Further, the approximated formula of the background emission was also derived. We analyzed the fluorescence intensities at various SD distances obtained by the experiments with and without a cylindrical ICG–milk target embedded at various depths in a ground beef phantom. The background signals determined the average effective attenuation coefficient, $0.16\;{\mathrm{mm}}^{-1}$, and a proportionality constant. Then, the experimental results for the fluorescence from the target at various depths validated the analytical expression of the fluorescence signal. The experimentally obtained variances of the detected signals confirmed that the fluctuation of the fluorescence signals was the fluctuation of the raw counting data with a large offset due to the ambient light. Using the nσ limit criterion, the depth detection limit of ∼30  mm was estimated with a short SD distance. The depth detection limits and the optimum SD distance were discussed in three cases. Finally, the clinical application of this technique was briefly discussed. This study demonstrated a novel variance analysis of the depth detection limit based on the analytical expressions with three factors, i.e., target fluorescence, background emission, and ambient light. The analysis here can apply to other setups in general.

## Supplementary Material



## Data Availability

Data underlying the results presented in this paper are not publicly available at this time but may be obtained from the authors upon reasonable request. See Secs. 1–4 in the Supplementary Material for supporting content.
