# Supplementary material for: Depth detection limit of a fluorescent object in tissue-like medium with background emission in continuous-wave measurements: a phantom study
Source: J Biomed Opt. 2024 Sep 2;29(9):097001. doi: 10.1117/1.JBO.29.9.097001 (PMC11368132; doi:10.1117/1.JBO.29.9.097001)
Supplement: Supplementary file 1 [file JBO_029_097001_SD001.pdf]

Depth detection limit of a fluorescent object in tissue-like medium with background emission in CW measurements – A phantom study: supplemental document

This supplemental document consists of four sections. The first section is dedicated to deriving Eqs. (1) and (2) in the main text. The second section explains the derivations of the approximated expressions in the main text, compares the approximated expression with the exact solution, and discusses the appropriate expression concerning the range of ρ. The third section shows a experimental result for the fluorescence intensity measurements with changing the concentration of ICG-milk solution. The last section considers the depth detection limit with some different conditions.

1 Derivation of the homogeneous background emission

Here, we derive Eqs. (1) and (2) in the main text, extending the result in the reference [S1] ([17] in the main text) to more general cases. At first, consider the fluorescence intensity detected at a detector position, $\mathbf{x}_\mathrm{d}$, when the excitation light of the power density, $I_\mathrm{x}(\mathbf{x}_\mathrm{s})$, is injected at a source position, $\mathbf{x}_\mathrm{s}$. The fluorescence intensity at $\mathbf{x}_\mathrm{d}$ consists of the fluorescence intensities, $F(s)$, corresponding to the optical path with the optical pathlength of s among all the optical paths between $\mathbf{x}_\mathrm{s}$ and $\mathbf{x}_\mathrm{d}$. The optical path consists of the excitation light propagating from $\mathbf{x}_\mathrm{s}$ to a position $\mathbf{x}$, where the fluorescence is generated, and the fluorescence light propagating from $\mathbf{x}$ to $\mathbf{x}_\mathrm{d}$, as shown in Fig. S1.1. For the fluorescence generated by the excitation light from a tiny volume of $\Delta\mathbf{x}$ at $\mathbf{x}$, the fluorescence intensity detected at $\mathbf{x}_\mathrm{d}$ is expressed by

$$\int_0^s d\sigma\, \gamma_\mathrm{f}\mu_\mathrm{af}(\mathbf{x})\Phi_\mathrm{m}(\mathbf{x}_\mathrm{d}, s-\sigma; \mathbf{x})\Phi_\mathrm{x}(\mathbf{x}, \sigma; \mathbf{x}_\mathrm{s})I_\mathrm{x}(\mathbf{x}_\mathrm{s})\Delta\mathbf{x},$$

where γ_f and $\mu_\mathrm{af}(\mathbf{x})$ are the quantum efficiency and the absorption coefficient of the fluorophores, respectively, and $\Phi_\mathrm{x}(\mathbf{x}_2, s; \mathbf{x}_1)$ and $\Phi_\mathrm{m}(\mathbf{x}_2, s; \mathbf{x}_1)$ are the propagation probabilities from $\mathbf{x}_1$ to $\mathbf{x}_2$ with a pathlength of s for the excitation and fluorescence light denoted by the subscripts x and m, respectively. σ is an arbitrary pathlength between $\mathbf{x}_\mathrm{s}$ and $\mathbf{x}$ along the path with the pathlength of s, which satisfies $\sigma \in [0, s]$.

The fluorescence intensity, $F(s)$, is given by the sum of the contribution of the fluorescence with the pathlength of s from all the position, $\mathbf{x}$, in the medium of Ω, yielding

$$F(s) = \int_\Omega d\mathbf{x} \int_0^s d\sigma\, \gamma_\mathrm{f}\mu_\mathrm{af}(\mathbf{x})\Phi_\mathrm{m}(\mathbf{x}_\mathrm{d}, s-\sigma; \mathbf{x})\Phi_\mathrm{x}(\mathbf{x}, \sigma; \mathbf{x}_\mathrm{s})I_\mathrm{x}(\mathbf{x}_\mathrm{s}). \tag{S1.1}$$

Eq. (S1.1) is the general form of the concept described in the paper by Patterson and Pogue in 1994[S1]. Since the time-of-flight measurement can determine the pathlength, this expression is equivalent to the solution of time-domain measurements with the zero-fluorescence lifetime. Integrating the fluorescence intensity, $F(s)$, overall pathlengths gives the total fluorescence intensity at $\mathbf{x}_\mathrm{d}$, leading to the following,

$$F(\mathbf{x}_\mathrm{d}; \mathbf{x}_\mathrm{s}) = \int_0^\infty ds \int_\Omega d\mathbf{x} \int_0^s d\sigma\, \gamma_\mathrm{f}\mu_\mathrm{af}(\mathbf{x})\Phi_\mathrm{m}(\mathbf{x}_\mathrm{d}, s-\sigma; \mathbf{x})\Phi_\mathrm{x}(\mathbf{x}, \sigma; \mathbf{x}_\mathrm{s})I_\mathrm{x}(\mathbf{x}_\mathrm{s}). \tag{S1.2}$$

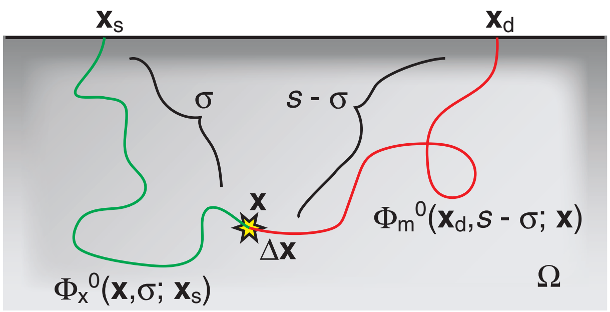

Figure S1.1: Schematic of an optical path with the optical pathlength of s between $\mathbf{x}_\mathrm{s}$ to $\mathbf{x}_\mathrm{d}$ via $\mathbf{x}$, where the fluroescence generated.

Since the convolution inside Eq. (S1.2) becomes a product of the infinite integrals of $\Phi_{\rm x}$ and $\Phi_{\rm m}$ with respect to the pathlength, the general form of Eq. (6) in the main text is obtained. It is worth noting that the discussion here is independent of the propagation models, such as the diffusion and radiative transfer equations and the geometry of the medium.

Suppose a homogeneous scattering medium with a homogeneous distribution of a fluorophore. We assume the so-called microscopic Beer-Lambert law for $\Phi_{\rm x}$ and $\Phi_{\rm m}$ as

$$\Phi_{\rm x}(\mathbf{x}_2, s; \mathbf{x}_1) = \Phi^0_{\rm x}(\mathbf{x}_2, s; \mathbf{x}_1) e^{-\mu_{\rm ax} s}, \tag{S1.3}$$

$$\Phi_{\rm m}(\mathbf{x}_2, s; \mathbf{x}_1) = \Phi^0_{\rm m}(\mathbf{x}_2, s; \mathbf{x}_1) e^{-\mu_{\rm am} s}, \tag{S1.4}$$

where $\mu_{\rm ax}$ and $\mu_{\rm am}$ are the absorption coefficient for the excitation and fluorescence light, respectively, $\Phi^0_{\rm x}$ and $\Phi^0_{\rm m}$ are the intensities of the excitation and fluorescence light in the medium without absorption, respectively. $\mu_{\rm af}(\mathbf{x})$ is replaced by a constant, $\mu_{\rm f}$, due to the homogeneous distribution of the fluorophore in the medium. Then, substituting Eqs. (S1.3) and (S1.4) into Eq. (S1.2) gives the following,

$$F(\mathbf{x}_{\rm d}; \mathbf{x}_{\rm s}) = \gamma_{\rm f} \mu_{\rm f} \int_0^\infty ds \int_\Omega d\mathbf{x} \int_0^s d\sigma\, \Phi^0_{\rm m}(\mathbf{x}_{\rm d}, s-\sigma; \mathbf{x}) e^{-\mu_{\rm am}(s-\sigma)} \Phi^0_{\rm x}(\mathbf{x}, \sigma; \mathbf{x}_{\rm s}) e^{-\mu_{\rm ax}\sigma} I_{\rm x}(\mathbf{x}_{\rm s}). \tag{S1.5}$$

The difference between $\Phi^0_{\rm x}$ and $\Phi^0_{\rm m}$ is caused by the wavelength dependent optical properties, such as the scattering and refractive index. We define the difference as $\delta\Phi^0_{\rm mx} = \Phi^0_{\rm m} - \Phi^0_{\rm x}$, and change the order of the integral for $\mathbf{x}$ an σ of Eq. (S1.5), yielding

$$\begin{aligned} F(\mathbf{x}_{\rm d}; \mathbf{x}_{\rm s}) = \gamma_{\rm f} \mu_{\rm f} \int_0^\infty ds \int_0^s d\sigma\, e^{-\mu_{\rm am}(s-\sigma)} e^{-\mu_{\rm ax}\sigma} \int_\Omega d\mathbf{x} \left[\Phi^0_{\rm x}(\mathbf{x}_{\rm d}, s-\sigma; \mathbf{x}) \Phi^0_{\rm x}(\mathbf{x}, \sigma; \mathbf{x}_{\rm s}) \right. \\ \left. + \delta\Phi^0_{\rm mx}(\mathbf{x}_{\rm d}, s-\sigma; \mathbf{x}) \Phi^0_{\rm x}(\mathbf{x}, \sigma; \mathbf{x}_{\rm s}) \right] I_{\rm x}(\mathbf{x}_{\rm s}). \end{aligned} \tag{S1.6}$$

The first integrand of the space integral is the joint probability from $\mathbf{x}_{\rm s}$ to $\mathbf{x}_{\rm d}$ via $\mathbf{x}$ where the pathlength, s, is divided into two pathlengths of σ and $s - \sigma$. By integrating the first integrand over all the space, the resultant integral becomes equal to the probability from $\mathbf{x}_{\rm s}$ to $\mathbf{x}_{\rm d}$ with the pathlength of s, namely,

$$\Phi^0_{\rm x}(\mathbf{x}_{\rm d}, s; \mathbf{x}_{\rm s}) = \int_\Omega d\mathbf{x}\, \Phi^0_{\rm x}(\mathbf{x}_{\rm d}, s-\sigma; \mathbf{x}) \Phi^0_{\rm x}(\mathbf{x}, \sigma; \mathbf{x}_{\rm s}).$$

Eventually, Eq. (S1.6) can be reduced to

$$\begin{aligned} F(\mathbf{x}_{\rm d}; \mathbf{x}_{\rm s}) = \gamma_{\rm f} \mu_{\rm f} \int_0^\infty ds \left[\frac{e^{-\mu_{\rm am} s} - e^{-\mu_{\rm ax} s}}{\mu_{\rm ax} - \mu_{\rm am}} \Phi^0_{\rm x}(\mathbf{x}_{\rm d}, s; \mathbf{x}_{\rm s}) \right. \\ \left. + \int_0^s d\sigma\, e^{-\mu_{\rm am}(s-\sigma)} e^{-\mu_{\rm ax}\sigma} \int_\Omega d\mathbf{x} \delta\Phi^0_{\rm mx}(\mathbf{x}_{\rm d}, s-\sigma; \mathbf{x}) \Phi^0_{\rm x}(\mathbf{x}, \sigma; \mathbf{x}_{\rm s}) \right] I_{\rm x}(\mathbf{x}_{\rm s}). \end{aligned} \tag{S1.7}$$

Finally, using Eqs. (S1.3) and (S1.4) and $\delta\Phi^0_{\rm mx} = \Phi^0_{\rm m} - \Phi^0_{\rm x}$, Eq. (S1.7) is reduced to the following,

$$\begin{aligned} F(\mathbf{x}_{\rm d}; \mathbf{x}_{\rm s}) = \frac{\gamma_{\rm f} \mu_{\rm f}}{\mu_{\rm ax} - \mu_{\rm am}} \int_0^\infty ds [\Phi_{\rm m}(\mathbf{x}_{\rm d}, s; \mathbf{x}_{\rm s}) - \Phi_{\rm x}(\mathbf{x}_{\rm d}, s; \mathbf{x}_{\rm s}) - e^{-\mu_{\rm am} s} \delta\Phi^0_{\rm mx}(\mathbf{x}_{\rm d}, s; \mathbf{x}_{\rm s})] I_{\rm x}(\mathbf{x}_{\rm s}) \\ + \gamma_{\rm f} \mu_{\rm f} \int_0^\infty ds \int_0^s d\sigma \int_\Omega d\mathbf{x}\, e^{-\mu_{\rm am}(s-\sigma)} \delta\Phi^0_{\rm mx}(\mathbf{x}_{\rm d}, s-\sigma; \mathbf{x}) \Phi_{\rm x}(\mathbf{x}, \sigma; \mathbf{x}_{\rm s}) I_{\rm x}(\mathbf{x}_{\rm s}) \end{aligned} \tag{S1.8}$$

The integral of $\Phi_x(\mathbf{x}_{\rm d}, s; \mathbf{x}_{\rm s})$ with respect to s is the probability of the excitation light at the detector position, $\mathbf{x}_{\rm d}$, when the light is injected at $\mathbf{x}_{\rm s}$, and that of Φ_m is for the case of the fluorescence light. Now, the excitation light intensity at $\mathbf{x}_{\rm d}$ is denoted by $R_{\rm x}$. Similarly, the light intensity in the case of the light at fluorescence wavelength injected at $\mathbf{x}_{\rm s}$ is denoted by $R_{\rm m}$. Noting $R_{\rm x} = \int_0^\infty ds \Phi_{\rm x}(\mathbf{x}_{\rm d}, s; \mathbf{x}_{\rm s})$ and $R_{\rm m} = \int_0^\infty ds \Phi_{\rm m}(\mathbf{x}_{\rm d}, s; \mathbf{x}_{\rm s})$, Eq. (S1.8) can be expressed by a simple expression as

$$F(\mathbf{x}_{\rm d}; \mathbf{x}_{\rm s}) = \frac{\gamma_{\rm f} \mu_{\rm f}}{\mu_{\rm ax} - \mu_{\rm am}} (R_{\rm m} - R_{\rm x}) I_{\rm x}(\mathbf{x}_{\rm s}) + \delta F, \tag{S1.9}$$

where δF is the remainder due to the differences in the optical properties other than the absorption between the excitation and fluorescence wavelengths in the medium and expressed as

$$\delta F = \gamma_{\rm f} \mu_{\rm f} \left[\int_\Omega d\mathbf{x}\, \delta\bar{\Phi}_{\rm mx}(\mathbf{x}_{\rm d}, \mathbf{x}) \bar{\Phi}_{\rm x}(\mathbf{x}; \mathbf{x}_{\rm s}) - \frac{1}{\mu_{\rm ax} - \mu_{\rm am}} \delta\bar{\Phi}_{\rm mx}(\mathbf{x}_{\rm d}, \mathbf{x}_{\rm s}) \right] I_{\rm x}(\mathbf{x}_{\rm s}), \tag{S1.10}$$

where $\delta\bar{\Phi}_{\rm mx}(\mathbf{x}_2, \mathbf{x}_1) = \int_0^\infty ds \delta\Phi^0_{\rm mx}(\mathbf{x}_2, s; \mathbf{x}_1) \exp(-\mu_{\rm m} s)$ and $\bar{\Phi}_{\rm x}(\mathbf{x}_2, \mathbf{x}_1) = \int_0^\infty ds \Phi_{\rm x}(\mathbf{x}_2, s; \mathbf{x}_1)$. Here, we used a relationship that the infinite integral of a convolution integral becomes a product of the infinite integrals of the integrands. Eq. (S1.9) does not directly depend on the propagation model and holds even for a non-scattering medium. In our particular case, we employ the diffusion model for the light propagation in a semi-infinite (a half-space) homogeneous medium. The measurements are conducted on the boundary, and resultantly, the measured fluorescence intensity only depends on the distance, ρ, between the source and the detector. We assume that the medium is illuminated by the unit intensity of the excitation light so that $I_{\rm x}(\mathbf{x}_{\rm s}) = 1$. Then, we substituted the analytical solution of the diffusion equation under the extrapolated boundary condition based on the partial current boundary condition into Eq. (S1.9) and Eq.(2) in the main text is obtained by neglecting the differences in the optical properties between the excitation and fluorescence light.

[S1] M.S. Patterson and B.W. Pogue, Appl.Opt. **33**. 1963–1974 (1994). [doi:10.1364/AO.33.001963]

2 The approximated expression of the background emission

2.1 Derivation of the approximated expression of the background emission

To derive an approximated expression to Eq. (1) in the main text, let us introduce the following Taylor expansion,

$$\frac{\exp[-\bar{\mu}\rho(1+\epsilon)^{1/2}]}{\rho(1+\epsilon)^{1/2}} = \frac{\exp(-\bar{\mu}\rho)}{\rho} - \frac{(1+\bar{\mu}\rho)\exp(-\bar{\mu}\rho)}{2\rho}\epsilon + \frac{1}{2!}\frac{(1+\bar{\mu}\rho+\bar{\mu}^2\rho^2)\exp(-\bar{\mu}\rho)}{4\rho}\epsilon^2 + O(\epsilon^3), \tag{S2.1}$$

when $\epsilon \ll 1$. Applying up to the first order term of the expansion, Eq. (S2.1), to R_y (y = x or m) expressed by Eq. (2), Eq. (1) is approximated as

$$F_\mathrm{B}(\rho)/\tilde{A} \sim \frac{(1+\bar{\mu}_\mathrm{m}\rho)\exp(-\bar{\mu}_\mathrm{m}\rho) - (1+\bar{\mu}_\mathrm{x}\rho)\exp(-\bar{\mu}_\mathrm{x}\rho)}{2\rho}(\epsilon_2-\epsilon_1), \tag{S2.2}$$

$$= \frac{\exp[-(\bar{\mu}_\mathrm{m}+\bar{\mu}_\mathrm{x})\rho/2]}{2\rho} \times \left[(1+\bar{\mu}_\mathrm{m}\rho)\exp\left(-\frac{\bar{\mu}_\mathrm{m}-\bar{\mu}_\mathrm{x}}{2}\rho\right) - (1+\bar{\mu}_\mathrm{x}\rho)\exp\left(-\frac{\bar{\mu}_\mathrm{x}-\bar{\mu}_\mathrm{m}}{2}\rho\right)\right](\epsilon_2-\epsilon_1), \tag{S2.3}$$

where $\epsilon_1 = z_0^2/\rho^2$, $\epsilon_2 = (z_0+2z_\mathrm{e})^2/\rho^2$ and $\tilde{A} = \alpha_\mathrm{B}/[8\pi AD(\mu_\mathrm{ax}-\mu_\mathrm{am})]$. Since $\epsilon_1 < \epsilon_2$, the approximation is valid when $\epsilon_2 \ll 1$. In other words, Eq. (S2.2) holds when the SD distance, ρ, is much larger than the scattering length $\mu_\mathrm{s}'^{-1}$ because $z_0 + 2z_\mathrm{e} = (1+4A/3)\mu_\mathrm{s}'^{-1}$.

Further approximation, we consider two cases of $|(\bar{\mu}_\mathrm{m}-\bar{\mu}_\mathrm{x})\rho| \ll 1$ (Case S1) and $|(\bar{\mu}_\mathrm{m}-\bar{\mu}_\mathrm{x})\rho| \gg 1$ (Case S2). Case S1 corresponds to the case that the difference of the effective attenuation coefficients (EACs), $|\bar{\mu}_\mathrm{x}-\bar{\mu}_\mathrm{m}|$, is much smaller than ρ^{-1}. On the other hand, Case S2 means that $|\bar{\mu}_\mathrm{m}-\bar{\mu}_\mathrm{x}|$ is much larger than ρ^{-1}.

For Case S1, expanding the second and third exponential terms in Eq. (S2.3) leads a further approximated expression as,

$$\text{(S2.3)} = \frac{\exp[-(\bar{\mu}_\mathrm{m}+\bar{\mu}_\mathrm{x})\rho/2]}{2\rho}\left[\frac{\bar{\mu}_\mathrm{x}^2-\bar{\mu}_\mathrm{m}^2}{2}\rho^2 + O((|\bar{\mu}_\mathrm{x}-\bar{\mu}_\mathrm{m}|\rho)^3)\right](\epsilon_2-\epsilon_1), \tag{S2.4}$$

$$\sim \frac{\exp[-(\bar{\mu}_\mathrm{m}+\bar{\mu}_\mathrm{x})\rho/2]}{4\rho}(\bar{\mu}_\mathrm{x}^2-\bar{\mu}_\mathrm{m}^2)[4z_\mathrm{e}(z_0+z_\mathrm{e})], \tag{S2.5}$$

and, using $\bar{\mu}_\mathrm{x}^2-\bar{\mu}_\mathrm{m}^2 = 3\mu_\mathrm{s}'(\mu_\mathrm{ax}-\mu_\mathrm{am})$, $3\mu_\mathrm{s}' = 1/D$, $z_0 = 3D$ and $z_\mathrm{e} = 2AD$, Eq. (S2.5) is simplified to,

$$F_\mathrm{B}(\rho) \sim \frac{\alpha_\mathrm{B}(3+2A)}{4\pi}\frac{\exp[-(\bar{\mu}_\mathrm{m}+\bar{\mu}_\mathrm{x})\rho/2]}{\rho} = \frac{\alpha_\mathrm{B}(3+2A)}{4\pi}\frac{\exp(-\bar{\mu}\rho)}{\rho}, \tag{S2.6}$$

where $\bar{\mu} = (\bar{\mu}_\mathrm{x}+\bar{\mu}_\mathrm{m})/2$.

For Case S2, $|(\bar{\mu}_\mathrm{m}-\bar{\mu}_\mathrm{x})\rho| \gg 1$ leads

$$\text{(S2.3)} = \frac{(1+\bar{\mu}_\mathrm{m}\rho)\exp(-\bar{\mu}_\mathrm{m}\rho)}{2\rho}\left\{1 - \frac{1+\bar{\mu}_\mathrm{x}\rho}{1+\bar{\mu}_\mathrm{m}\rho}\exp[-(\bar{\mu}_\mathrm{x}-\bar{\mu}_\mathrm{m})\rho]\right\}(\epsilon_2-\epsilon_1)$$

$$\sim \frac{\bar{\mu}_\mathrm{m}\exp(-\bar{\mu}_\mathrm{m}\rho)}{2}(\epsilon_2-\epsilon_1) = \frac{2\bar{\mu}_\mathrm{m}\exp(-\bar{\mu}_\mathrm{m}\rho)[z_\mathrm{e}(z_0+z_\mathrm{e})]}{\rho^2}, \tag{S2.7}$$

when $\bar{\mu}_\mathrm{m} < \bar{\mu}_\mathrm{x}$ because $(1+\bar{\mu}_\mathrm{x}\rho)/(1+\bar{\mu}_\mathrm{m}\rho)\exp[-(\bar{\mu}_\mathrm{x}-\bar{\mu}_\mathrm{m})\rho]$ exponentially approaches 0. When $\bar{\mu}_\mathrm{m} > \bar{\mu}_\mathrm{x}$, exchanging roles of $\bar{\mu}_\mathrm{m}$ and $\bar{\mu}_\mathrm{x}$ in Eq. (S2.7) leads similarly the approximated expression. Then, we have the approximated expression of $F_\mathrm{B}(\rho)$ as,

$$F_\mathrm{B}(\rho) \sim \frac{\alpha_\mathrm{B}(3+2A)}{2\pi}\frac{\mu_{\mathrm{a}y}}{|\bar{\mu}_\mathrm{m}-\bar{\mu}_\mathrm{x}|}\frac{\exp(-\bar{\mu}_y\rho)}{\rho^2} \tag{S2.8}$$

where $\bar{\mu}_y = \min(\bar{\mu}_\mathrm{x}, \bar{\mu}_\mathrm{m})$ and $\bar{\mu}_{\mathrm{a}y} = \min(\bar{\mu}_\mathrm{ax}, \bar{\mu}_\mathrm{am})$.

2.2 Numerical validation of the approximated expressions

Some numerical comparisons between the exact calculation and its approximations for $F_{\rm B}(\rho)$ are shown in Fig. S2.2 to validate the approximated expressions. The open circles and the crosses show the exact expressions of Eq. (1) for Case A $(\mu_{\rm ax}, \mu_{\rm am}) = (0.011, 0.01)$ and Case B $(0.011, 0.02)$ (the unit is mm^{-1}), respectively. Other parameters are as $\mu_{\rm s}' = 1\,{\rm mm}^{-1}$, $n = 1.37$ and $\alpha_{\rm B} = 1$. The curves show the calculations of the above approximations.

In Case A, where $\mu_{\rm ax}$ and $\mu_{\rm am}$ are close together, the approximations by Eqs. (S2.3) and (S2.6) are visually indistinguishable and agree well with the exact expression except for $\rho < 10 - 15$ mm. However, the approximation by Eq. (S2.8) is significantly larger than the exact expression but gradually approaching the exact one (pink solid curve). In Case B, where the difference between $\mu_{\rm ax}$ and $\mu_{\rm am}$ becomes large, the approximated expression of Eq. (S2.8) is much closer to the exact expression except at short ρ (pink broken curve).

The relative errors defined as $F_{\rm B}^{\rm approx}/F_{\rm B}^{\rm exact} - 1$ are plotted in Fig. S2.2 (b), where $F_{\rm B}^{\rm exact}$ and $F_{\rm B}^{\rm approx}$ denote the exact $F_{\rm B}$ of Eq. (1) and the approximated $F_{\rm B}$ of Eqs. (S2.3) or (S2.6) or (S2.8), respectively. The solid and broken curves show the errors of the approximations in Case A and Case B, respectively. In Case A, the difference of the relative errors between Eq. (S2.3) and (S2.6) is visually indistinguishable. Both relative errors are less than 10% when $\rho > 15$ mm. In Case B, the errors of Eq. (S2.3) become slightly more significant than in Case A but are very similar. The result for Eq. (S2.3) is reasonable because the first step approximation does not directly depend on the absorption coefficients $\mu_{\rm ax}$ and $\mu_{\rm am}$. In Case B, the errors of Eq. (S2.6) are similar to those in Case A at small ρ. However, the error gradually increases with increasing $\rho (> 25$ mm). The errors of Eq. (S2.8) are significant both in Case A and Case B, although those in Case B approach 0 much faster with increasing ρ.

2.3 Discussion on the approximations and the SD distance

The approximated expressions as Eqs. (S2.5) and (S2.8) are derived by the two-step expansion under $\epsilon_2 = (z_0 + 2z_{\rm e})^2/\rho^2 \ll 1$ for the first step and $|\bar{\mu}_{\rm x} - \bar{\mu}_{\rm m}|\rho \gg 1$ or $|\bar{\mu}_{\rm x} - \bar{\mu}_{\rm m}|\rho \ll 1$ for the second step. In this section, we discuss these expressions under realistic ones for biological tissue measurements.

For many biological tissues, the reduced scattering coefficient, $\mu_{\rm s}'$, is of the order of magnitude of 1 mm^{-1} in a near-infrared wavelength range, and the absorption coefficient, $\mu_{\rm a}$, is about two orders of magnitude smaller than $\mu_{\rm s}'$. We can also expect that the variations of $\mu_{\rm s}'$ and $\mu_{\rm a}$ are much smaller than these values when the excitation and detection light wavelengths are close together. For example, many previous studies employed $\mu_{\rm s}' = 1$ mm^{-1}, $\mu_{\rm a} = 0.01$ mm^{-1}, and a refractive index of $n = 1.37$ as typical values for biological tissues, yielding $z_0 + 2z_{\rm e} \sim 5$ mm and $\bar{\mu} = 0.17$ mm^{-1}. The first condition $(z_0 + 2z_{\rm e})^2/\rho^2 \ll 1$ leads $\rho \gg 5$ mm. It is worth noting that the first condition ϵ_2 inversely proportional to the square of ρ, and the values of ρ ranging 15–30 mm in our current experiment lead to $\epsilon_2 < 1/9$, which mostly satisfies the first condition.

The example of Case B in the numerical validation assumes a slightly extreme case with a large difference between $\mu_{\rm ax}$ and $\mu_{\rm am}$, i.e., $\bar{\mu}_{\rm x}$ and $\bar{\mu}_{\rm m}$, to visualize the effect of the difference on the numerical results as shown in Fig. S2.2. Now, we consider a much smaller difference between EACs ($\bar{\mu}_{\rm x}$ and $\bar{\mu}_{\rm m}$) as a realistic example. If the difference of EACs is 5%, the second condition in Case S1 is written as $\rho \ll 118$ mm. The realistic range of ρ is usually smaller than 40 mm, and this range satisfies $\rho \ll 118$ mm to some extent. In contrast, the second condition in Case S2 requests $\rho \gg 118$ mm, and this range of ρ is usually outside of the measurable condition.

The performance of the approximated expression of Eq. (S2.6) is similar to that of Eq. (S2.3), as shown in Fig. S2.2. Consequently, Eq. (S2.3) is a simple and useful expression for $F_{\rm B}$ in realistic conditions. In addition, the expression can be applied to estimate the average EACs, $\bar{\mu}$, by measuring $F_{\rm B}$ with varying ρ. Therefore, we employ Eq. (S2.6) for the analysis of $F_{\rm B}$.

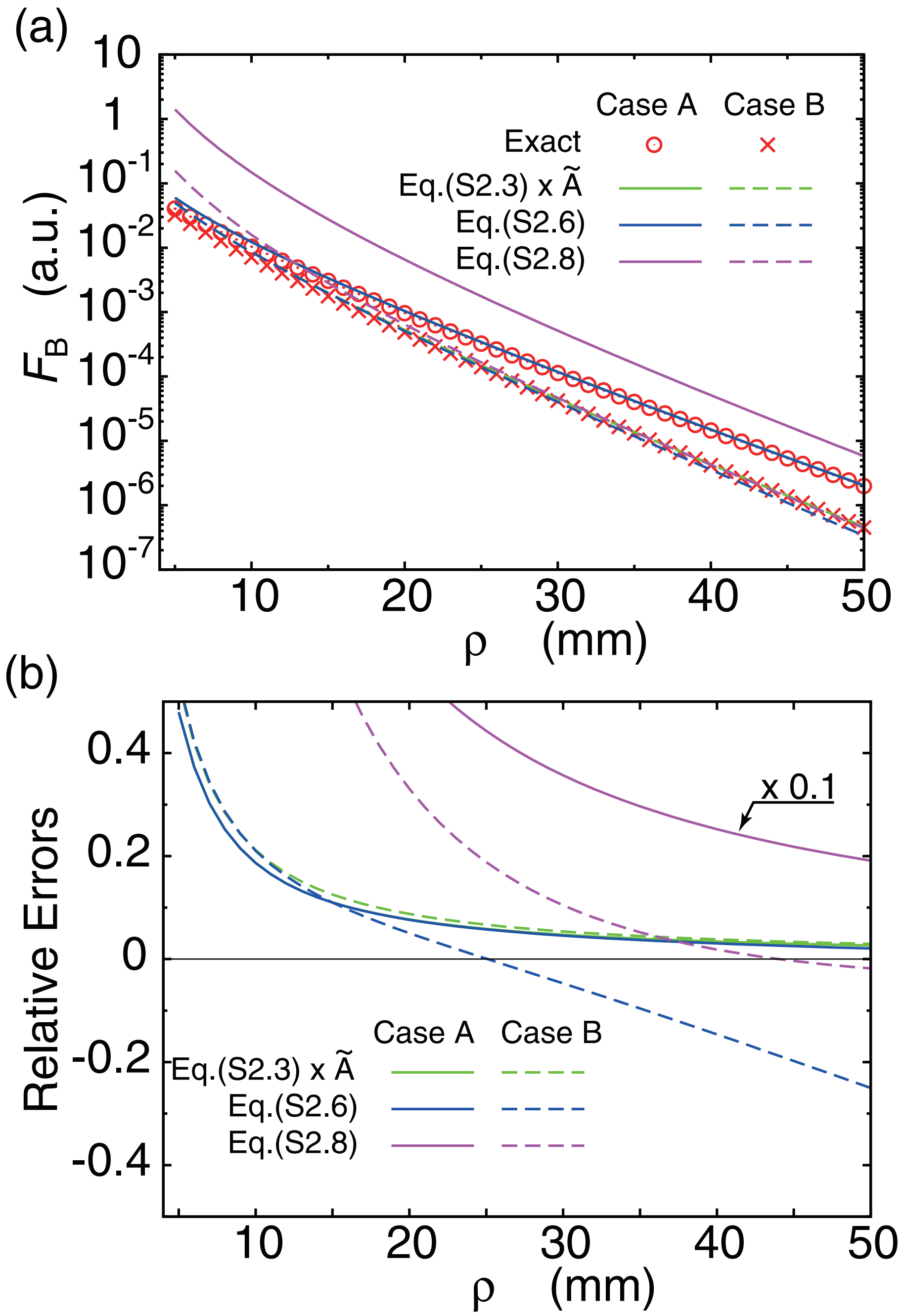

Figure S2.2: The background fluorescence intensity F_{B} against the SD distance ρ (a) and the relative error of the approximations (b). The open circles and crosses show the exact experssion of Eq.(1) with $(\mu_{\mathrm{ax}}, \mu_{\mathrm{am}}) = (0.011, 0.01)$ (Case A) and $(0.011, 0.02)$ (Case B), respectively. The solid and dashed curves show the results of the approximations. The relative errors of Case A with Eq. (S2.8) are scaled by 0.1 (purple solid curve).

3 Concentration dependent fluorescence intensity

We measured the fluorescence intensity by changing the ICG concentration of the ICG-milk solution. The experiment was essentially the same as the one in the main text. The differences from the main experiment were using a fixed SD distance of 30 mm and inserting a thin plastic film between the meat phantom and the source and detector fibers. The excitation power was fixed to 20 mW. The ICG-milk solution was encapsulated in a thin plastic cylinder (6 mm in diameter and 20 mm in length).

Figure S3.3 shows the experimental results of the measured fluorescence intensities as a function of the ICG concentration with varying z_{T}. The measured fluorescence intensities reach their maximum at about 1 μM of the ICG concentration, and the peak positions slightly move to higher concentrations as z_{T} increases. We, therefore, chose a concentration of about 1 μM for the experiment in the main text.

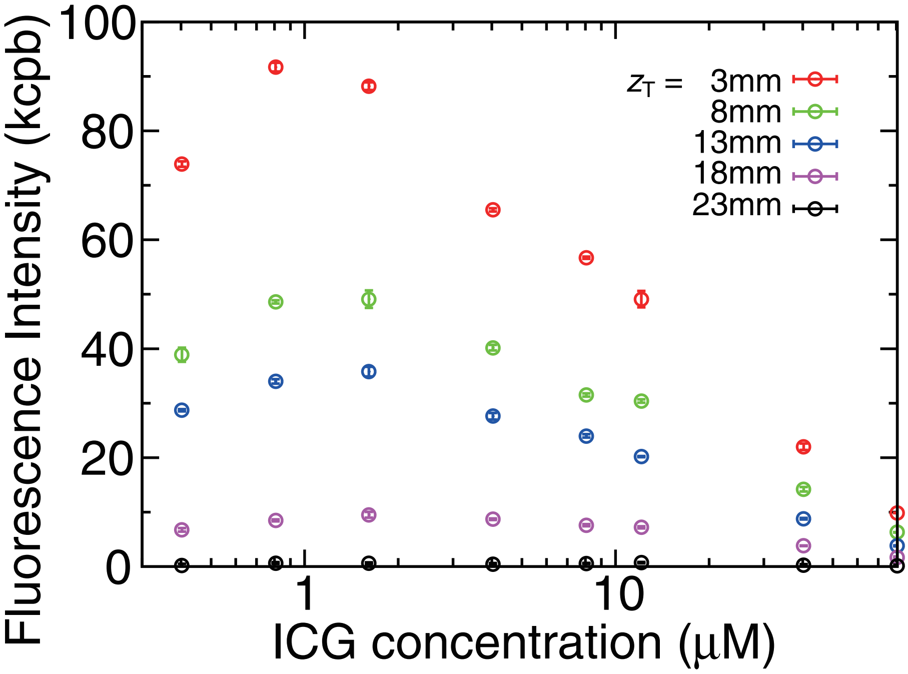

Figure S3.3: Fluorescence intensities with changing the ICG concentration in the ICG-milk solution from 0.4 μM to 80 μM at ρ = 30 mm while varying the depth of the ICG-milk capsule, z_{T}, from 3 mm to 25 mm, indicated by colors.

4 Depth detection limits under some conditions different from those in the main text

In this paper, ground beef was used as the phantom medium, and particular values of the laser power, the concentration of ICG, and the optical properties were chosen in the measurements and simulations. In applications to human subjects, the optical properties of the human tissue may be different from those of the phantom, and the different laser power and concentration of ICG may be applicable in the measurements. In the following, we show some simulation results to understand how these conditions affect the depth detection limit.

The simulations were conducted under three different conditions for comparison with the results, as shown in Fig. 6 in the main text: square root two times larger the EAC (doubled absorption coefficients of the medium, μ_{ax} and μ_{am}), doubled I_{x} (the excitation power), and doubled μ_{T} (the absorption coefficient of the target proportional to the ICG concentration). For doubled μ_{T}, the inner filter effect and the photophysical property of ICG were not considered, resulting doubled fluorescence intensity from the target. The results are shown in Fig. S4.4 with different colors and line styles (the figure on the right is an enlarged portion of the figure on the left).

The dotted curves for square root two times larger EAC (doubled μ_{ax} and μ_{am}) indicate that the depth detection limits, $z_{\mathrm{T}}^{\mathrm{lim}}$, for all four cases of this experiment and Cases 1–3 become 6–7 mm shallower than those in the phantom experiments shown by the solid curves. However, the optimum SD distances change only slightly. Since the difference in the absorption coefficients causes the change of the target and background fluorescence intensities through the EACs, $\bar{\mu}_{\mathrm{x}}$ and $\bar{\mu}_{\mathrm{m}}$, as Eqs. (3)–(5) in the main text, these results suggest that the differences in the EAC of the tissue among subjects affect the depth detection limit. The preliminary measurement results of the EAC for the human subjects indicate a variation in the EAC caused by the differences in the absorption and scattering coefficients, suggesting that $z_{\mathrm{T}}^{\mathrm{lim}}$ for humans may vary from person to person. On the other hand, these simulation results indicate that the optimum SD distance can be held constant for all the human subjects.

The dashed curves for the double I_{x} indicate that $z_{\mathrm{T}}^{\mathrm{lim}}$ values increase about 2 mm from the solid curves, meaning that $z_{\mathrm{T}}^{\mathrm{lim}}$ is slightly improved by increasing I_{x}. The dash-dotted curves for double μ_{T} are almost the same as the dashed curves for doubled I_{x} except for Case 3 (blue curves) and except the range of ρ shorter than about 12 mm for other cases. Increasing excitation power and ICG concentration can improve the depth detection limit. However, the differences in the above conditions do not affect the optimum SD distance.

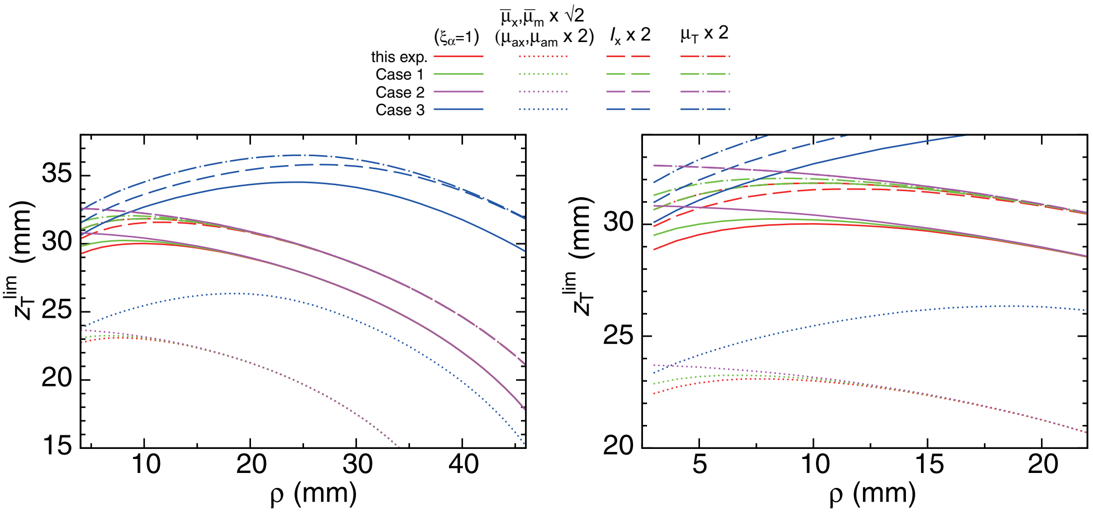

Figure S4.4: The depth detection limit, $z_{\mathrm{T}}^{\mathrm{lim}}$, as a function of the SD distance, ρ, with three different conditions from those in Fig. 6: square root two times larger EAC (doubled μ_{ax} and μ_{am}), doubled I_{x}, and doubled μ_{T} indicated by the dotted, dashed, and dash-dotted curves, respectively. The solid curves are the results at $\xi_{\alpha} = 1$ in Fig.6. The figure on the right enlarges a portion of the figure on the left for the short ρ. The dashed and dash-dotted curves in cases of this experiment, Case 1, and Case 2 are indistinguishable except for very short ρ less than about 12 mm as shown in the figure on the right.
